# Cardiac hemodynamics and ventricular stiffness of sea-run cherry salmon (*Oncorhynchus masou masou*) differ critically from those of landlocked masu salmon

**DOI:** 10.1371/journal.pone.0267264

**Published:** 2022-11-04

**Authors:** Yuu Usui, Misaki Kimoto, Akira Hanashima, Ken Hashimoto, Satoshi Mohri

**Affiliations:** First Department of Physiology, Kawasaki Medical School, Kurashiki, Japan; Mayo Clinic, UNITED STATES

## Abstract

Ventricular diastolic mechanical properties are important determinants of cardiac function and are optimized by changes in cardiac structure and physical properties. *Oncorhynchus masou masou* is an anadromous migratory fish of the Salmonidae family, and several ecological studies on it have been conducted; however, the cardiac functions of the fish are not well known. Therefore, we investigated ventricular diastolic function in landlocked (masu salmon) and sea-run (cherry salmon) types at 29–30 months post fertilization. Pulsed-wave Doppler echocardiography showed that the atrioventricular inflow waveforms of cherry salmon were biphasic with early diastolic filling and atrial contraction, whereas those of masu salmon were monophasic with atrial contraction. In addition, end-diastolic pressure–volume relationship analysis revealed that the dilatability per unit myocardial mass of the ventricle in cherry salmon was significantly suppressed compared to that in masu salmon, suggesting that the ventricle of the cherry salmon was relatively stiffer (relative ventricular stiffness index; *p* = 0.0263). Contrastingly, the extensibility of cardiomyocytes, characterized by the expression pattern of Connectin isoforms in their ventricles, was similar in both types. Histological analysis showed that the percentage of the collagen accumulation area in the compact layer of cherry salmon increased compared with that of the masu salmon, which may contribute to ventricle stiffness. Although the heart mass of cherry salmon was about 11-fold greater than that of masu salmon, there was no difference in the morphology of the isolated cardiomyocytes, suggesting that the heart of the cherry salmon grows by cardiomyocyte proliferation, but not cell hypertrophy. The cardiac physiological function of the teleosts varies with differences in their developmental processes and life history. Our multidimensional analysis of the *O*. *masou* heart may provide a clue to the process by which the heart acquires a biphasic blood-filling pattern, i.e., a ventricular diastolic suction.

## Introduction

Ventricular stiffness, myocardial relaxation, and atrial contraction are important for diastolic ventricular filling, a major determinant of cardiac output [1]. Ventricular stiffness is adjusted to suit the lifestyle of an individual and increases with age and growth [2, 3] by varying muscle mass, architecture, and geometry of the chambers [4]. Investigations and comparisons of the ventricular diastolic properties of individuals within a species at different developmental stages and body weights are, therefore, crucial to advance our understanding of the physiological adaptations of the heart.

Ventricular diastolic functions at the organ and tissue level are evaluated by echocardiography and ventricular pressure–volume relationship analysis. For example, Doppler echocardiography shows blood inflow waveforms that determine the speed and direction of blood transfer from the atria to the ventricles resulting from the pressure gradient between them [5]. Assessment of mechanical properties has also been used since early studies of cardiac mechanics [6–8]. End-diastolic pressure–volume relationship (EDPVR) analysis has been developed as one of the important concepts for interpreting cardiac mechanics and is established as a valid evaluation method for ventricular stiffness for both *in vivo* and *ex vivo* studies [4, 9–12]. EDPVRs are non-linear; the steeper the slope (dV/dP) the stiffer the ventricle. Clinical studies of EDPVR have reported diastolic dysfunction with increasing ventricular stiffness resulting from hypertension and aortic stenosis [13, 14]. When assessing the stiffness of ventricles of different sizes, their relative ventricular stiffness has also been evaluated by normalization [15, 16]. Collagen accumulation, changes in collagen types, and inflammation were the distinguishing factors of stiffer ventricles [17–20].

The passive tension of cardiomyocytes is one of the determinants of ventricular filling dynamics as it provides resistance to expansion of the ventricle lumen. Connectin, encoded by *ttn*, is a protein comprising sarcomeres, and as a biological spring, it functions to generate tension forces in accordance with sarcomere lengths [21–24]. The elastic potential of Connectin depends on the splicing of N2A and/or N2B spring segments, number of PEVK spring segment repeats, and phosphorylation levels of the protein [24–30]. Mammalian cardiomyocytes co-express the Connectin isoforms N2BA (3,700 kDa and compliant) and N2B (3,000 kDa and stiff) [31–34]. The expandability of cells changes according to the ratio of these isoforms [30, 35–37].

Teleosts possess a heart with four compartments: a sinus venosus, atrium, ventricle, and bulbus arteriosus (S1A and S1B Fig) [38–40]. Each compartment is separated by a valve. Most teleost ventricles are composed of two histologically distinct myocardial layers: a compact layer and a spongy layer [41–44]. The former is circumferentially arranged from dense cardiomyocytes as the ventricular wall under the epicardium, while the latter has cardiomyocytes arranged in a network, and is found in the luminal region of the ventricles. Venous blood from the atrium flows into the intertrabecular space. Based on the histological findings for compact layer thickness and the distribution patterns of the coronary vessels, fish ventricles are classified into four types (types I–IV) [44–46].

The family Salmonidae is extremely diverse and includes 11 genera and at least 70 extant species [47]. Most species change their habitats with migratory behaviors and have adapted to both freshwater and seawater environments. Owing to these characteristics, their cardiovascular systems have been investigated as useful models for physiology [41, 48–52]. A triangular pyramidal ventricle is common in salmonids (e.g., Atlantic salmon [*Salmo salar*], sockeye salmon [*Oncorhynchus nerka*], and rainbow trout [*Oncorhynchus mykiss*]) [42, 53]. As the nutrient vessels at the ventricle are confined to the compact layer, salmon ventricles are classified as type II. In addition, this ventricle type is believed to be an effective pump as it can transfer blood at higher heart rates than saccular and tubular ventricles [54, 55]. The salmonid compact layer thickens as it grows [56, 57]. Exercise, mechanical stress, and hormones also remodel the salmonid ventricle [58–64], and thermal changes induce collagen accumulation and increase ventricular stiffness [65, 66].

*Oncorhynchus masou masou* has two predominant life history types: landlocked (masu salmon) and sea-run (cherry salmon) (S1C Fig) [67, 68]. These fish have remarkably different skin patterns and body sizes. Transient increases in the secretion of hormones (e.g., the thyroid hormone, growth hormone, cortisol, adrenocortical hormones, and sex hormones) are involved in the smolt and anadromous processes [67–72]. While there have been many studies on the ecology of *O*. *masou*, to the best of our knowledge there have been no studies to date on its cardiac functions. We therefore investigated the possible differences in the ventricular diastolic functions of masu and cherry salmon. To compare the diastolic function of their hearts, we observed their atrioventricular inflows using pulsed-wave Doppler echocardiography and evaluated their passive ventricular mechanical properties by pressure–volume analysis *in vivo* and *ex vivo*. We also compared their heart histology, cardiomyocyte morphology, and the expression patterns of Connectin isoforms.

## Materials and methods

### Experimental approval

All experiments were performed following the guidelines and approved protocols for animal care and use (approval number for animal use: 20–147) of the animal experiment committee at Kawasaki Medical School.

### Masu and cherry salmon

Twenty-one juvenile fish, six months post fertilization (mpf), were purchased from Azuma Yougyojou Y.K. (Gunma, Japan). They were maintained for one week in 8-L freshwater tanks, with 10 fish per tank. A total of 30 masu salmon (*O*. *masou* landlocked type) 29–30 mpf (25 fish) and 34 mpf (5 fish) were purchased from Utanogawa Yamame Yougyojou (Yamaguchi, Japan), were maintained in 70-L freshwater tanks (5 or 10 fish per tank), and used in experiments within 1 week of their arrival. Eleven cherry salmon (*O*. *masou* sea-run type) at 29–30 mpf were purchased from Wakao-Suisan Co., Ltd. (Hyogo, Japan), maintained in 200-L seawater tanks with a salinity of 35 g/L (1.023 specific gravity) [73], at two or three fish per tank. For the cherry salmon, *in vivo* experiments were performed within two days of their transport. All fish tanks were maintained at 10°C with a 14-/10-h light/dark cycle, fed a commercial diet (Remix, Meito Suien Co., Ltd., Aichi, Japan, Cat# M-450). Fish were randomly selected for this study as sex could not be specified at the time of purchase.

### Echocardiography and electrocardiography

Echocardiography in pulsed-wave Doppler mode and electrocardiography were recorded using an Aplio 300 system (Toshiba Medical System Corp., Tochigi Japan) with a 14-MHz transducer and three electrodes. Mild anesthetic induction by MS-222 (50 mg/L, Sigma-Aldrich, Merck KGaA, Darmstadt, Germany) [74] was used to treat five masu salmon and two cherry salmon until there was minimal gill movement. The anesthetized fish were placed upside down in the tank. The ultrasound transducer probe was placed on their heart positions, and the detection depth and brightness were adjusted for each fish (S2A Fig). The waveforms of atrioventricular inflows were observed immediately after ventricular outflow recordings because it is impossible to measure these flows simultaneously with a single probe. From the pulse-wave Doppler images, ventricular outflow times and velocities were calculated using Fiji software version 2.3.0 [75]. Intervals between the end of the ventricular outflow and the onset of the first atrioventricular inflow were calculated by subtracting the average time of ventricular outflow from each time from the peak of the R wave to the onset of atrioventricular inflow. The changes in the potential across the body surface by electrical excitation of the heart were measured by clipping electrodes to their pectoral and pelvic fins (S2A Fig). Aeration was performed during the measurements. After recording, all fish were euthanized using 250 mg/L MS-222 for organ sampling.

### *In vivo* atrial and ventricular pressure measurements

Five masu salmon 34 mpf were anesthetized using 50 mg/L MS-222 and were placed upside down in the tank. A Single Transducer Set, DTX Plus DT-4812 (Argon Medical Devices, Inc., Plano, TX, USA), with needles was used to measure the pressure. Using an echocardiographic live image, a 25-G needle was inserted from the ventral side into the ventricle and a 27-G needle was inserted under the operculum into the atrium. The ventricular pressure tracing data were used to mediate the pressure recording interface (Power Lab 8/30, ADInstruments Pty Ltd., Dunedin, New Zealand) and were digitized at 200 Hz using LabChart 7 software (ADInstruments) and recorded. After recording, all fish were euthanized using 250 mg/L MS-222.

### *Ex vivo* ventricular pressure–volume analysis

Four masu salmon and three cherry salmon were euthanized as described above, after which their hearts were removed. A catheter was inserted into the ventricles through the atrioventricular valve and fixed by ligation. Fluids in the ventricular lumen were washed using 10 U/mL heparin FUJIFILM Wako Pure Chemical Corp., Osaka, Japan) and 10 mM 2,3-butanedione monoxime (BDM; FUJIFILM Wako Pure Chemical Corp.) in Ca^2+^- and Mg^2+^-free phosphate-buffered saline (PBS; Takara Bio Inc., Kusatsu, Japan). The aortic valve was then ligated. To measure the pressure–volume relationship, saline solution was injected into the ventricles using an infusion pump (Terumo Corp., Tokyo, Japan). The ventricular pressure data were recorded at 400 Hz with a multielectrode conductance catheter using the LabChart 7 software. The following exponential equation was fit to the EDPVR data:

$$P_{ressure}=A+Be^{C}{}^{V},$$ (1)

where A, B, and *C* are constants that describe the ventricular exponential pressure-normalized volume property; constant A indicates the intercept on the pressure axis (A = -B), and *C* the index of the ventricular stiffness. V is the ventricular volume [4]. When comparing the ventricular stiffness in hearts of different sizes, V was normalized by the ventricular mass to calculate the stiffness per unit of ventricular mass and described by the following equation:

$$V=v_{t}m^{-1},$$ (2)

where *v*_*t*_ was the total volume of saline infused into the ventricle at time *t*, *v*_0_ = 0, and *m* was the ventricular mass [15].

### Tissue staining

Masu and cherry salmon hearts were fixed in 4% paraformaldehyde (PFA; Sigma-Aldrich, Merck) at 4°C for 48 h and embedded in paraffin blocks. The mounted glass slides with 5-μm-thick heart sections were immersed in Clear Plus (FALMA Corp., Tokyo, Japan) followed by 100%, 95%, and 70% ethanol solutions for deparaffinization, and finally rinsed with water.

### Elastica van Gieson staining

The heart sections were immersed in Maeda’s resorcinol fuchsin stain solution (Muto Pure Chemicals Co., Ltd.) for 1 h and washed with 100% ethanol. Heart sections (5-μm thick) were mounted on glass slides and immersed in Weigert’s iron hematoxylin solution and washed with Milli-Q water. The specimens were then immersed in Picro–Sirius Red solution (ScyTek Laboratories, Inc., West Logan, UT, USA) for 15 min, air-dried, and rinsed with xylene. After staining, the tissue sections were dehydrated in 70%, 95%, and 100% ethanol solutions and ethanol/xylene mixed solution, immersed in xylene four times, and sealed using a mounting medium (Muto Pure Chemicals Co., Ltd., Tokyo, Japan). The sections were observed under a bright-field microscope BX53, (Olympus, Tokyo, Japan) using the cellSens software version 1.18 (Olympus) and BZ-X700 microscope (Keyence, Osaka, Japan). The collagen fiber content in the compact layer and cardiomyocyte density were measured using the Fiji software version 2.3.0 [75].

### Hematoxylin and eosin staining

The heart sections were immersed in Hansen’s hematoxylin solution (Sigma-Aldrich, Merck) for 5 min, followed by washing with water. To define the nuclei, sections were immersed in 0.2% hydrochloric acid–70% ethanol solution for 1 min, and then immersed in eosin Y solution (Millipore, Merck) for 30 s. After staining, the tissue sections were dehydrated in 70%, 95%, and 100% ethanol solutions and ethanol/xylene mixed solution, immersed in xylene four times, and sealed using a mounting medium. The sections were observed under a bright-field microscope BX53, using the cellSens software version 1.18 and BZ-X700 microscope. The collagen fiber content in the compact layer and cardiomyocyte density were measured using the Fiji software version 2.3.0 [75].

### Cardiomyocyte isolation and primary cultures

For isolation and primary cultures of the cardiomyocytes, we used a previously described protocol for zebrafish [76]. Two masu salmon and two cherry salmon 29–30 mpf were euthanized, their ventricles collected, and their lumens washed by injecting heparin buffer (10 U/mL heparin in PBS). The ventricles were dissected into 3-mm^2^ columns and shaken at 800 rpm for 90–120 min at 30°C with 750 μL digestion buffer (12.5 μM CaCl_2_, 5 mg/mL collagenase type II [Thermo Fisher Scientific Inc., Waltham, MA, USA], 5 mg/mL collagenase type IV [Thermo Fisher Scientific Inc.], 10 mM HEPES, 30 mM taurine, 5.5 mM glucose, and 10 mM BDM). Next, the cell suspension was filtered through a 100-μm nylon cell strainer, and 1 mL CaCl_2_ buffer (12.5 μM CaCl_2_, 10% fetal bovine serum [FBS; HyClone, Cytiva, Marlborough, MA, USA, Lot#15N353], 10 mM HEPES, 30 mM taurine, 5.5 mM glucose, and 10 mM BDM) was added and the cell suspension centrifuged at 200 × *g* for 5 min at 4°C. After discarding the supernatant, 1 mL of CaCl_2_ buffer was added and the mixture was centrifuged at 200 × *g* for 5 min at 4°C. Isolated cells were resuspended using the culture media (Eagle’s minimum essential medium with 4.5 g/L glucose, 2 mM L-glutamine, 1 mM sodium pyruvate, and phenol red [FUJIFILM Wako Pure Chemical Corp.], 5 mM BDM, 5% FBS, and 100 U/mL penicillin-streptomycin [Thermo Fisher Scientific Inc.]) and seeded on a glass-bottom dish (Iwaki Co. Ltd., Tokyo, Japan) with 0.01% poly-L-lysine (Millipore, Merck) coating. Primary cells were cultured at 25°C under 5% CO_2_. Twenty-four hours after incubation, the primary cells were observed using a BZ-X700 fluorescence microscope. The longitudinal length and area of the cardiomyocytes were measured using the Fiji software version 2.3.0 [75].

### Immunostaining

Primary cardiomyocytes were fixed in 4% PFA at 4°C for 15 min. They were then permeabilized with 0.02% Triton X-100 in PBS for 15 min and blocked with 5% bovine serum albumin (Sigma-Aldrich, Merck) in PBS for 3 h. These cells were incubated with primary antibody, monoclonal anti-α-actinin (Sarcomeric) antibody produced in mouse (1:1000, Sigma-Aldrich, Merck, clone EA-53, Cat# A7811), at 4°C overnight, and subsequently stained with a secondary antibody, polyclonal goat anti-mouse IgG (H+L) cross-adsorbed secondary antibody, Alexa Fluor 488 (1:1000, Thermo Fisher Scientific Inc., Cat# A-11001), and Hoechst33342 (Tokyo Chemical Industry Co., Ltd., Tokyo, Japan, Cat# H342) for 1 h at 25°C. The sarcomeric structures and the number of nuclei in the cardiomyocytes were then examined using an FV1000 confocal laser scanning microscope system mounted on IX81 (Olympus).

### Sodium dodecyl sulfate-agarose gel electrophoresis and western blotting

The atria, ventricles, and bulbus arteriosus were separated from the hearts of the masu and cherry salmon 29–30 mpf. Each sample was homogenized using Polytron (Kinematica AG, Malters, Switzerland) in a sample buffer (8 M urea, 2 M thiourea, 3% sodium dodecyl sulfate [SDS], 75 mM dithiothreitol, 0.03% bromophenol blue, 0.05 M Tris-HCl [pH 6.8], and protease inhibitor cocktail [Thermo Fisher Scientific Inc.]), heated at 65°C for 10 min, centrifuged at 16,000 × *g* for 5 min at 4°C, and the supernatant was collected. Cleared lysates were loaded into wells of the SDS-agarose gels (1% SeaKem Gold Agarose [Lonza, Basel, Switzerland], 30% glycerol, and 1 × Tris/glycine/SDS [TG-SDS] buffer [Takara Bio Inc.]) and then electrophoresed in a running buffer (1 × TG-SDS buffer and 10 mM 2-mercaptoethanol) at 0.01 A for 90 min [77]. To avoid the gel from sliding off the gel plate during manipulation, the SDS-agarose gel was stacked and fixed on a 1-cm-high acrylamide plug gel (12% acrylamide, 10% glycerol, 0.5 M Tris-HCl [pH 9.0], 0.0015% N,N,N′,N′-tetramethylethylenediamine, and 0.056% ammonium persulfate). The patterns of the band peaks on the Coomassie Brilliant Blue (CBB)-stained gels were scanned using the Gel Analyzer plugin of Fiji software version 2.3.0.

For western blotting, the electrophoresed proteins were transferred from SDS-agarose gel to a nitrocellulose membrane (Bio-Rad Laboratories, Inc., Hercules, CA, USA) with a semi-dry western blot system (Pierce Power Blotter, Thermo Fisher Scientific Inc.) at 25 V for 10 min. After blocking the membrane with Tris buffer saline containing 0.05% Tween 20 and 5% skim milk, the Connectin proteins in the heart tissue were identified using a polyclonal primary antibody, the C-terminus of chicken Connectin (1:1000, Pc72C) [78], and polyclonal goat anti-rabbit immunoglobulin/HRP (1:1000, DAKO Agilent Technologies, Inc., Palo Alto, CA, USA, Cat# P044801-2) as a secondary antibody. Connectin blots were detected using the chemiluminescence reagent Western Lightning ECL Pro (PerkinElmer Co., Ltd., Waltham, MA, USA) and acquired using ImageQuant LAS 4000 (GE Healthcare, Chicago, IL, USA).

### Transmission electron microscopy

To observe the sarcomere structures in the cardiomyocytes using a transmission electron microscope (TEM), the fish hearts at 29–30 mpf were washed with 10 U/mL heparin and 10 mM BDM in PBS and fixed with 4% PFA and 2.5% glutaraldehyde in PBS for 1 h at 25°C. The fixatives were thoroughly washed with PBS. The samples were then post-fixed in 1% osmium tetroxide in PBS and immersed in 50%, 60%, 70%, 80%, 90%, 95%, 99%, and 100% ethanol consecutively for dehydration. Next, the samples were passed through propylene oxide, embedded in epoxy resin, and polymerized in an incubator for 72 h at 60°C. Sections that were 500 nm thick were produced from the resin block using an ultramicrotome and collected on glass slides. After reaching the desired section, 70-nm ultrathin sections were cut and placed on a single-hole grid coated with Formvar film. The grids were air-dried and stained with uranyl acetate and lead citrate. Electron micrographs were recorded using TEM JEM-1400 (JEOL Ltd., Tokyo, Japan), operated at 80 kV.

### Statistical analysis

The experimental results are presented as the mean ± standard deviation (SD); the number of samples used in each analysis is indicated in each figure legend. *P*-values were calculated using Student’s *t*-test or one-way analysis of variance (ANOVA) with Tukey’s multiple comparisons test and are indicated on graphs and text. The threshold for significant difference was set at a *p*-value of ≤ 0.05. GraphPad Prism8 software version 8.4.3 (GraphPad Software, San Diego, CA, USA) was used to conduct all statistical analyses.

## Results

### Evaluation of the ventricular hemodynamics

**Fig 1:**
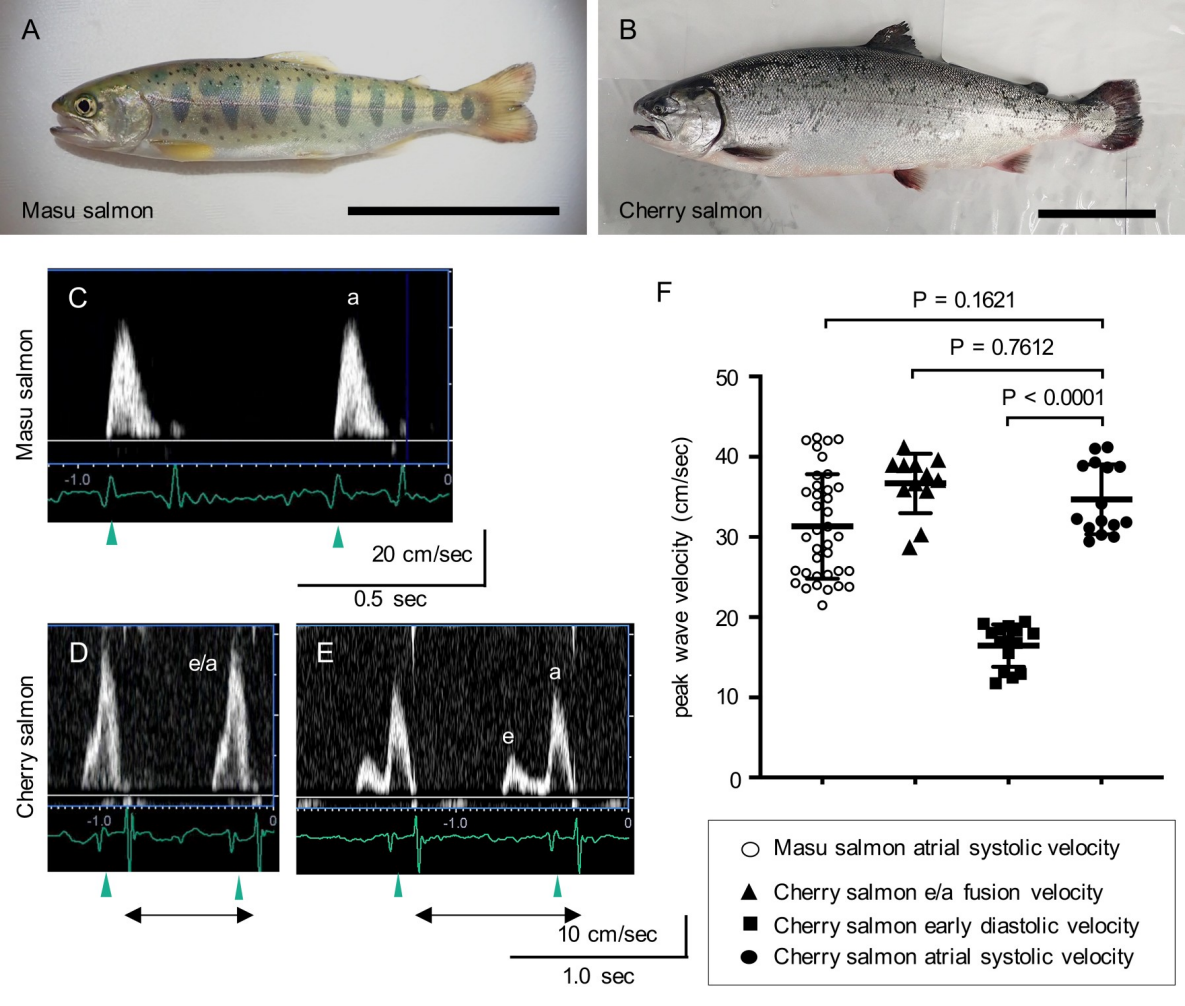
Recordings of the waveforms of the atrioventricular inflows in masu and cherry salmon using pulsed-wave echocardiography. (A, B) Representative *Oncorhynchus masou masou* pictures: (A) masu salmon at 29 months post fertilization, (B) cherry salmon reared in seawater for five months after being reared in freshwater for 24 months. Scale bars = 10 cm. (C, D, E) Representative velocity waveforms of atrioventricular inflows in the masu (C) and cherry salmon of monophasic (D) and biphasic patterns (E). Blood passing the atrioventricular valve was observed using pulsed-wave Doppler echocardiography simultaneously with electrocardiography. Green arrowheads indicate P waves. Black left–right double arrows indicate R-R intervals. a: atrial systolic velocity, e/a: fusion velocity of atrial systolic and early diastolic inflows, e: early diastolic velocity. (F) Peak wave velocity. ○: masu salmon atrial systolic velocity (31.3 ± 6.4 cm/s, N = 37 peaks from five fish), ▲: cherry salmon early diastolic waves and atrial systolic waves (e/a) fusion velocity (36.7 ± 3.6 cm/s, N = 12 peaks from two fish), ■: cherry salmon early diastolic velocity (16.5 ± 2.6 cm/s, N = 15 peaks from two fish), ●: cherry salmon atrial systolic velocity (34.7 ± 4.2 cm/s, N = 15 peaks from two fish). Lines and error bars indicate the mean ± standard deviation. Minimal data sets for atrioventricular velocities are found in S1 File.

At 29–30 mpf, the cherry salmon were found to be approximately 2.4 times bigger and 17 times heavier than the masu salmon (Fig 1A and 1B; Table 1). We also investigated their hemodynamics at this developmental stage. Pulsed-wave Doppler echocardiography and electrocardiography were simultaneously performed to observe their atrioventricular inflow in sinus rhythms. To set the pulsed-wave Doppler gate window downstream of the atrioventricular valve, a sagittal long-axis view including the atrium, ventricle, and bulbus arteriosus was acquired (S2B and S2C Fig; S1 and S2 Movies). Electrocardiography revealed regular P waves, QRS complexes, and T waves (S2D Fig).

**Table 1:** Body size and heart mass measurements (means ± standard deviation) for *Oncorhynchus masou masou*.

	N	Age (months)	Standard length (cm)	Full length (cm)	Body maximum height (cm)	Body weight	Heart mass	Heart mass (g) / Body weight (kg)
Juvenile	19	6	5.65 ± 0.40	6.65 ± 0.39	0.92 ± 0.10	1.49 ± 0.22 (g)	2.73 ± 0.27 (mg)	1.91±0.39
Masu salmon	20	29–30	18.90 ± 0.52	21.28 ± 0.62	4.16 ± 0.66	0.08 ± 0.25 (kg)	0.15 ± 0.02 (g)^†^	1.99±0.29^†^
Cherry salmon	11	29–30	45.46 ± 3.30	47.38 ± 3.60	11.20 ± 1.14	1.36 ± 0.37 (kg)	1.69 ± 0.19 (g)^††^	1.51±0.17^††^

^†^ N = 13.

^††^ N = 7.

Pulsed-wave Doppler echocardiography revealed that the atrioventricular inflow patterns in the masu salmon showed sequential single forward flow waveforms that were synchronized with the P waves, indicating atrial contraction (Fig 1C). The heart rate of masu salmon as recorded with echocardiography was 109 ± 12 beats/min (bpm, N = 5 fish) (S1 Table). In masu salmon #5, the time from the R wave to the end of ventricular outflow was 326.8 ± 1.0 ms (N = 4 beats) (S3A Fig). When the interval times for the onset of ventricular systole (R-R interval times) took 624 ms (short) and 958 ms (long), the time between the end of ventricular outflow and the appearance of atrioventricular inflow waveforms was estimated to be 160 ms and 396 ms, respectively (S3 Fig). Regardless of the duration of the R-R interval, the atrioventricular inflow waveforms showed a monophasic pattern; furthermore, the atrial pressure in the ventricular diastole of the masu salmon was the same as the ventricular pressure for a while after isovolumic relaxation and became higher than the ventricular pressure in the atrial systole (S4 Fig). Hence, the monophasic inflow waveforms in masu salmon were presumed to indicate atrial systolic velocity.

Interestingly, the forward flow waveforms began to appear in the atrioventricular inflow patterns of the cherry salmon, even before the P waves were recorded (Fig 1D). When the R-R interval took > 0.77 s, echography recorded clear biphasic waveforms (Fig 1E, S3 Fig). The low forward waveforms appeared before the P wave, and the high forward waveforms appeared after the P wave. The former and the latter were, therefore, presumed to be comparable to the early ventricular diastolic and atrial systolic velocities, respectively. In addition, the sequential monophasic waves in cherry salmon were likely to be the fusion of these two velocities (early ventricular diastolic and atrial systolic fusion velocity). The mean ± SD of the early diastolic velocity peak in the cherry salmon was 16.5 ± 2.6 cm/s, which was lower than the atrial systolic velocity (*p* < 0.0001, Fig 1F). The ratio of the early diastolic velocity/atrial systolic velocity was 0.48 ± 0.06 in the cherry salmon. In cherry salmon #1, the interval time from the R wave to the end of ventricular outflow was 352 ± 1 ms (N = 4 beats). Whether the R-R interval times were 720 ms (short), 772 ms (intermediate), or 908 ms (long), the times from the end of ventricular outflow until the appearance of atrioventricular inflow waveforms remained almost the same, at 92–96 ms (S3 Fig). Under MS-222 anesthesia, the heart rates of cherry salmon were 72 ± 5 bpm (N = 2 fish) and lower than that of masu salmon (*p* = 0.016, S1 Table).

### Analysis of ventricular stiffness

**Fig 2:**
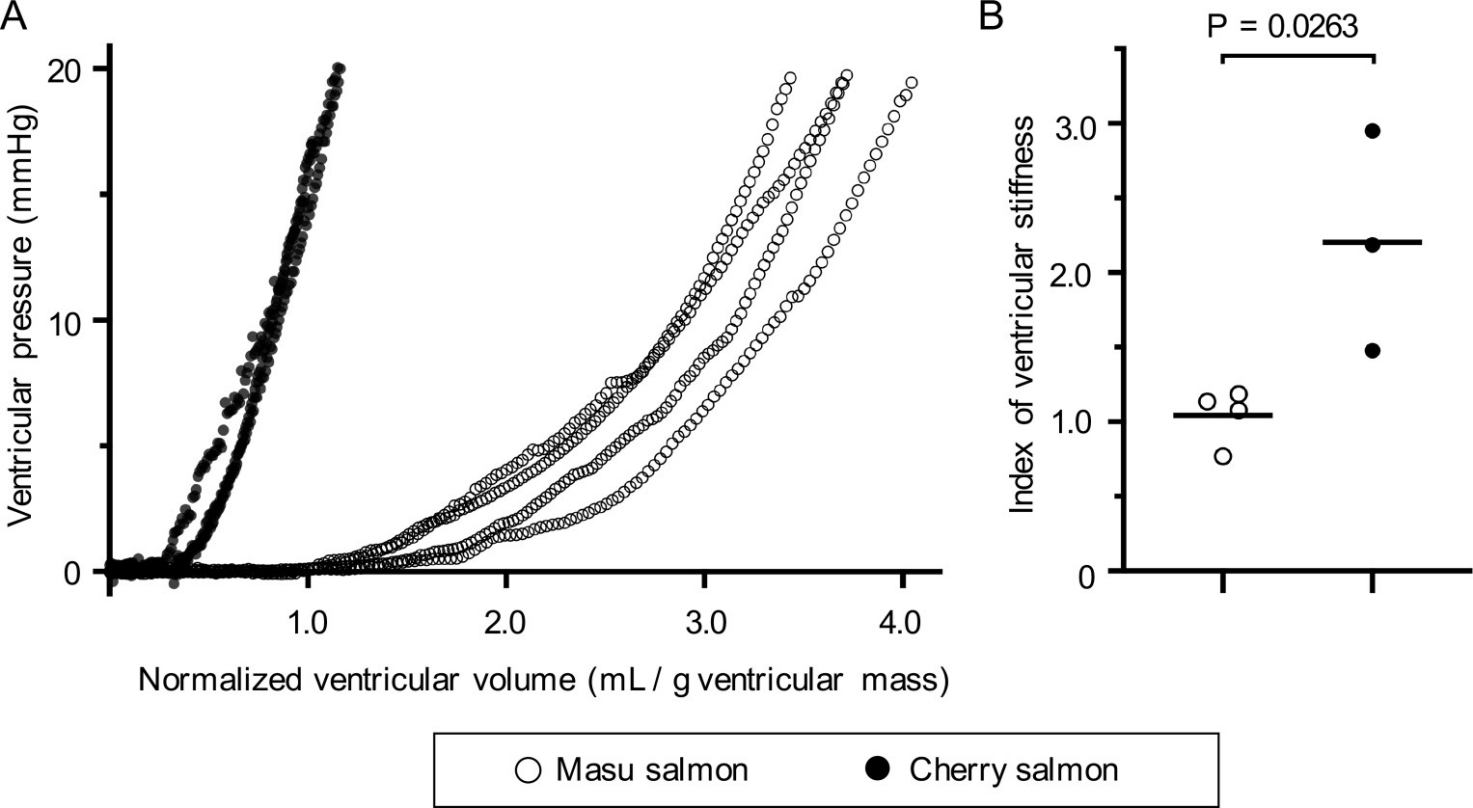
Relative ventricular stiffness of masu and cherry salmon. (A) The normalized end-diastolic pressure–volume relationship (EDPVR) at 30 mpf for masu and cherry salmon. The horizontal axis shows the ventricular volume normalized by the mass of the masu (N = 4) and cherry salmon ventricles (N = 3). The vertical axis shows the ventricular pressure. Ventricular mass is shown in S5B Fig. (B) Scores for ventricular stiffness in the masu and cherry salmon. The results of the EDPVR (A) were appended in Eq (1) to obtain the exponential *C*. Black horizontal lines in (B) indicate the means. ○: masu salmon, ●: cherry salmon.

To investigate ventricular stiffness, we performed the EDPVR analysis *ex vivo*. The interventricular pressure of masu salmon reached 20 mmHg in response to injection with 0.27–0.31 mL saline (S4A Fig), while that of cherry salmon reached ~20 mmHg in response to injection with 1.13–1.91 mL saline, and the smaller ventricles tended to show steeper EDPVR curves (S4 Fig). However, the normalized EDPVR curves of the cherry salmon shifted to the left were steeper compared to those of the masu salmon (Fig 2A). The indices of ventricular stiffness were 1.04 ± 0.19 in masu salmon and 2.21 ± 0.74 in cherry salmon, and the ventricles of cherry salmon were significantly stiffer than those of masu salmon based on the measurement of ventricular stiffness per unit myocardial mass (*p* = 0.02, Fig 2B).

### Morphological and histological analysis

**Fig 3:**
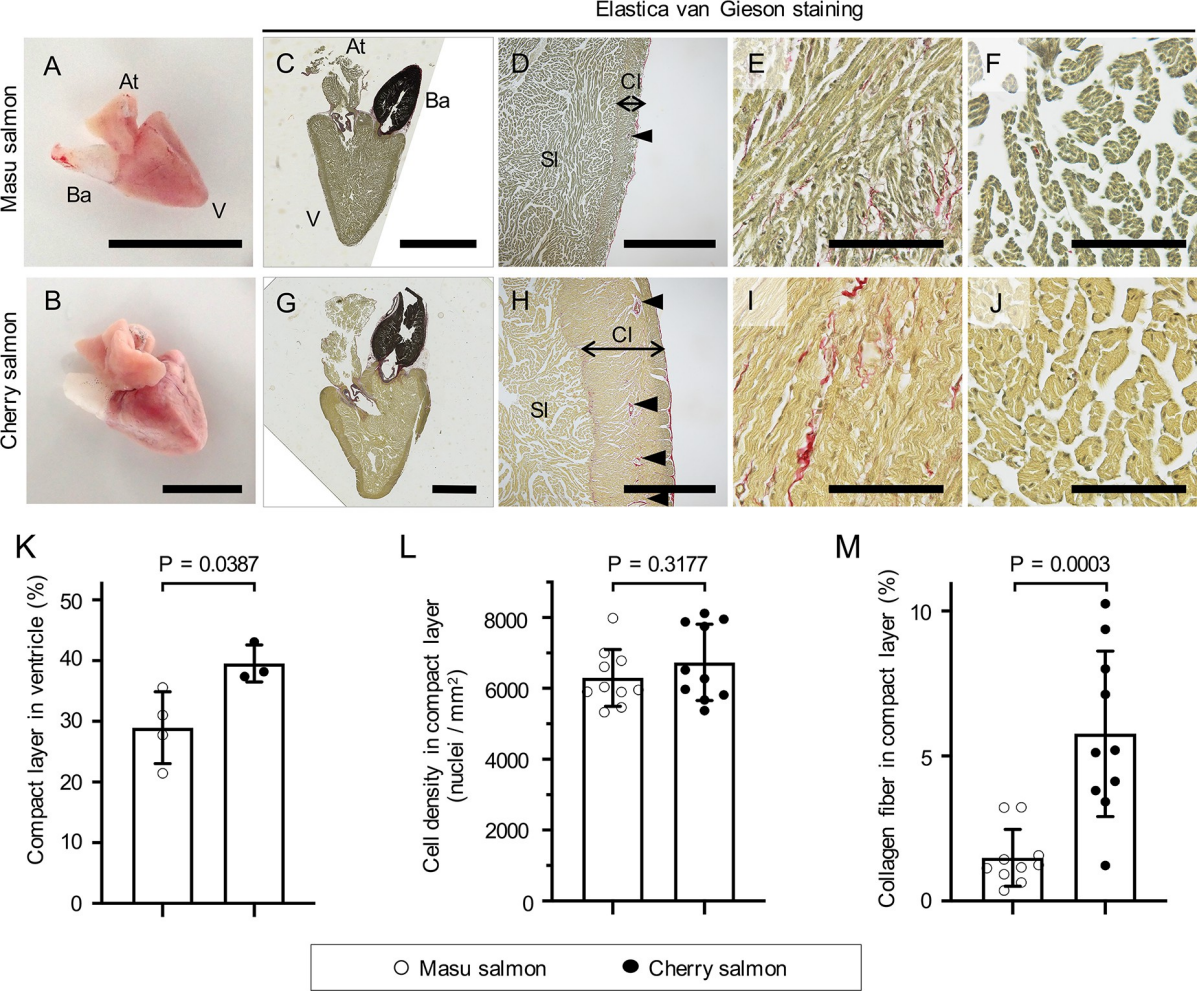
Histological analysis of masu and cherry salmon hearts. (A, B) Representative images of masu (A) and cherry (B) salmon hearts 29 mpf. At: atrium, V: ventricle, Ba: bulbus arteriosus. (C–J) Elastica van Gieson staining images of the sagittal sections of the hearts of masu and cherry salmon 29 mpf. (C, G) Images of the whole hearts. (D, H) Magnified images of the ventricles. Black double arrows indicate the thickness of the compact layer. Black arrowheads indicate coronary vessels. (E, I) Higher magnification images of the compact layers. (F, J) Higher magnification images of the spongy layers. Collagen fibers: bright red; cytoplasm: yellow; elastic fibers: purple-black; nuclei: dark black. Cl: compact layer, Sl: spongy layer. Scale bars = 1 cm in (A) and (B); 5 mm in (C) and (G); 1 mm in (D) and (H); 100 μm in (E), (I), (F), and (J). (K) Percentage compact layer in the sagittal ventricle sections. (L) The number of nuclei per yellow area in the compact layer. (M) The collagen fiber area percentage in the compact layer. Bar graphs and error bars indicate the means and standard deviations, respectively. ○: masu salmon, ●: cherry salmon. Minimal data sets of (K–M) are found in S3 File.

At 29–30 mpf, the ventricles of both fish displayed a pyramidal shape (Fig 3A and 3B). The heart mass of cherry salmon was approximately 11 times greater than that of masu salmon, although the heart mass relative to body weight was significantly greater for masu salmon than for cherry salmon (*p* = 0.006, Table 1). The juvenile ventricles at 6 mpf also exhibited pyramidal shapes (S1D and S1E Fig). The Elastica van Gieson-stained and hematoxylin and eosin-stained sections of masu and cherry salmon hearts (Fig 3C–3J and S6 Fig) showed that their ventricles had at least two layers of myocardium, i.e., a compact layer and a spongy layer (Fig 3C, 3D, 3G and 3H). The compact layer of cherry salmon had more coronary vessels compared with that in masu salmon (Fig 3D and 3H). Further, this layer was thicker in cherry salmon than that in masu salmon (Fig 3D and 3H), and the ratios of the compact layer area to the total ventricular area on sagittal heart slices were significantly different between masu (29.0% ± 5.1%) and cherry salmon (39.6% ± 2.5%; *p* = 0.039, Fig 3K). The cell density in the compact layer was 6297 ± 762 and 6734 ± 1021 nuclei/mm^2^ in masu and cherry salmon, respectively, and there was no significant difference between masu and cherry salmon (*p* = 0.318, Fig 3L).

The extracellular matrix is one of the parameters influencing tissue compliance. For example, elastin is an extracellular matrix that provides tissues with high levels of elasticity [79]. Bulbus arteriosus is an elastin-rich tissue [80] and stains purple–black with Elastica van Gieson staining (Fig 3C and 3G). The collagen fibers were tinted an intense red with Elastica van Gieson staining. Investigation of the heart sections revealed that the compact and spongy layers included collagen fibers (Fig 3E, 3F, 3I and 3J). The epicardium, the area around the coronary vessels, and the contact areas of the compact and spongy layers were found to be rich in collagen fibers (Fig 3D and 3H). The collagen fibers occupied 1.5% ± 0.9% of the compact layer in the ventricles from masu salmon and 5.8% ± 2.7% of the compact layer in the ventricles from cherry salmon, and the latter exhibited a significant accumulation of collagen fibers (*p* = 0.0003, Fig 3M).

**Fig 4:**
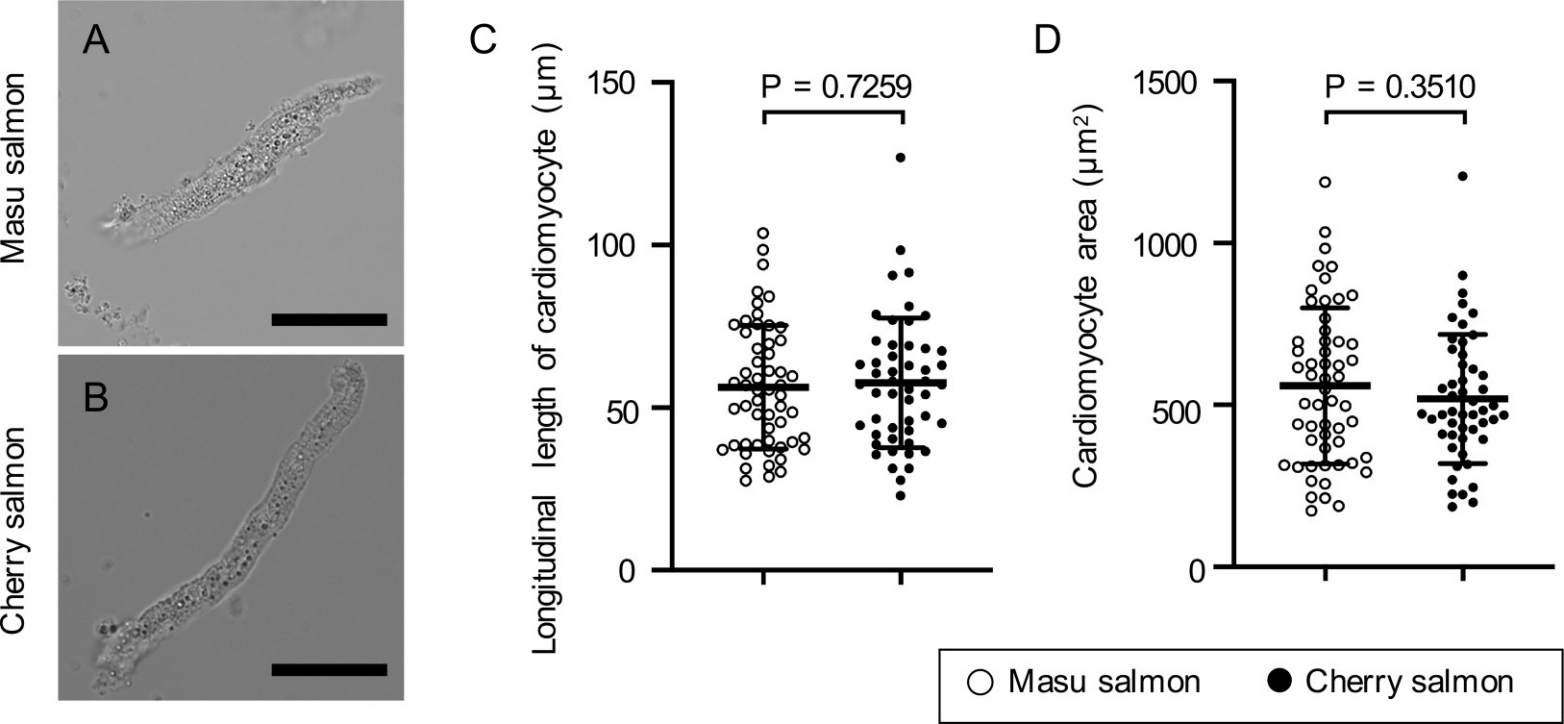
Morphology and sizes of the isolated cardiomyocytes. (A, B) Representative bright-field images of the primary cultured cardiomyocytes of masu and cherry salmon 29 mpf. Scale bars = 20 μm. (C) Length of the long axis of the isolated cardiomyocytes; masu salmon (56 ± 19 μm, N = 55), cherry salmon (58 ± 20 μm, N = 50). (D) Area of the isolated cardiomyocytes; masu salmon (559 ± 239 μm^2^, N = 55), cherry salmon (518 ± 197 μm^2^, N = 50). Lines and error bars indicate the mean ± standard deviation. ○: masu salmon, ●: cherry salmon. Actual measurements of cardiomyocyte morphology are shown in the S4 File.

To investigate whether cell hypertrophy is involved in heart growth, the cardiomyocytes of masu and cherry salmon were isolated, and their morphologies were analyzed. Most of the isolated cells that were attached to the dish showed spindle or rectangular shapes (Fig 4A and 4B). These cells were stained in a band pattern using the cardiomyocyte marker α-actinin 2 (S7A–S7D Fig). Smaller and round-shaped cells were negative for α-actinin 2 (S7E and S7F Fig). The longitudinal length and area of the cultured primary cardiomyocytes were not significantly different between masu (56 ± 19 μm and 559 ± 239 μm^2^) and cherry salmon (58 ± 20 μm and 518 ± 197 μm^2^; *p* = 0.7 and *p* = 0.4; Fig 4C and 4D, respectively). In these primary culture experiments, we found one binucleated cardiomyocyte in cherry salmon, and the rest of the cardiomyocytes were mononucleated (S8 Fig).

### Connectin expression patterns

**Fig 5:**
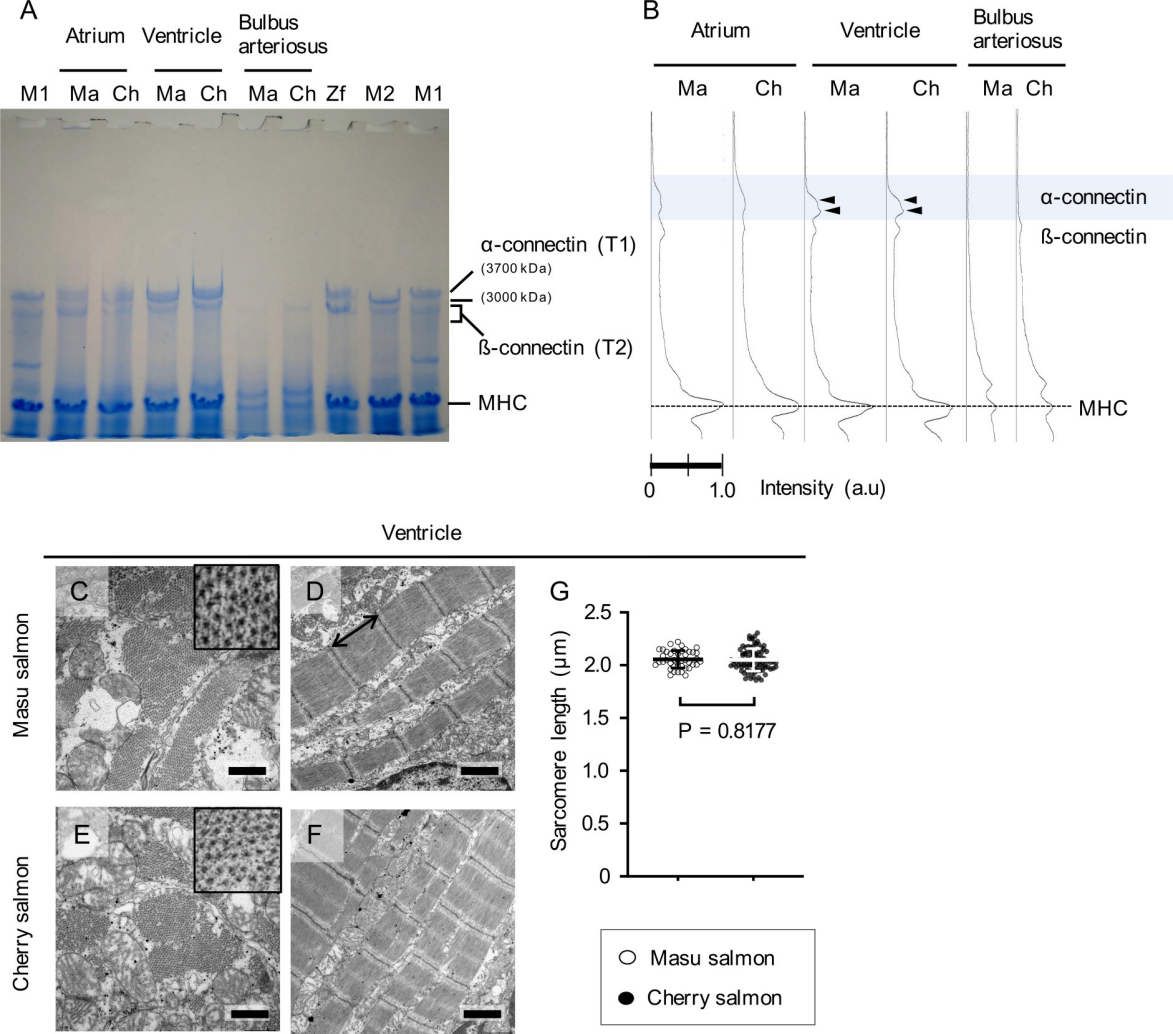
Molecular weights of the Connectin isoforms and sarcomere lengths in the hearts of masu and cherry salmon. (A) A representative CBB-stained gel image. Lanes 2–7 show the molecular weights of Connectin isoforms in the atrium, ventricle, and bulbus arteriosus of masu salmon (Ma) and cherry salmon (Ch). The following samples were used as molecular weight standards: a major Connectin isoform in zebrafish heart (Zf, 3500 kDa) in lane 8; Connectin N2A isoform from the skeletal muscles of mice (M1, 3700 kDa) in lanes 1 and 10; the Connectin N2B isoform expressed in the left ventricle of mice (M2, 3000 kDa) in lane 9. α-connectin (T1) and β-connectin (T2) indicate an intact Connectin and its degraded product, respectively. MHC indicates Myosin heavy chain (molecular weight ~220 kDa each), which was used as a loading control. (B) Electropherogram of lanes 2–7 of the CBB-stained gel in (A). Black arrowheads indicate the band peaks for the ventricles of masu and cherry salmon in the α-connectin zone. (C–F) Representative transmission electron microscopy images of the cross-sections and longitudinal sections in the ventricular sarcomeres of masu and cherry salmon 29 months post fertilization. Insets of (C, E) showing higher magnification images of the cross-sections of myofibril bundles. Scale bars = 500 nm in (C) and (E) and 1 μm in (D) and (F). (G) The ventricular sarcomere lengths of the z-line to z-line in the ventricles of masu salmon (2.06 ± 0.08 μm, N = 42) and cherry salmon (2.05 ± 0.11 μm, N = 90). A black double arrow indicates sarcomere length. Lines and error bars indicate the means ± standard deviations. ○: masu salmon, ●: cherry salmon. A raw data image of CBB-stained gel is found in S1 Raw images. Minimal data sets for (G) are found in S5 File.

The expression patterns of connectin isoforms affect ventricular diastolic function [30, 35–37]; these patterns are regulated by thyroid hormone in mammals [81]. In *O*. *masou*, increased secretion of this hormone induces downstream migratory behavior and smoltification [69, 72]. From these assumptions, we hypothesized that masu and cherry salmon exhibit different expression patterns for cardiac Connectin isoforms. To investigate the cardiac Connectin expression patterns, SDS-agarose gel electrophoresis was performed (Fig 5A). Band pattern analysis of the CBB-stained gel revealed the presence of two peaks in the α-connectin (T1) zone, indicating expression of intact Connectin in the ventricles (Fig 5B). Furthermore, western blotting with the anti-Connectin antibody [78] confirmed that these bands corresponded to Connectin (S9 Fig). These results suggest that *O*. *masou* at 29–30 mpf expressed Connectin (~3,700 kDa) in their ventricles and that there was no difference in the expression pattern of Connectin between masu and cherry salmon.

The sarcomere length of the ventricular myocardium did not vary significantly between the masu (2.06 ± 0.08 μm) and cherry salmon (2.05 ± 0.11 μm; *p* = 0.8, Fig 5C–5G). Similarly, the sarcomere length of the atrial myocardium also did not vary significantly between the masu (2.13 ± 0.16 μm) and cherry salmon (2.12 ± 0.11 μm; *p* = 0.7, S10A–S10E Fig). Moreover, microfilaments and collagen fibers were observed in the bulbus arteriosus of both the masu and cherry salmon (S10F–S10H Fig) [82].

## Discussion

### Differing ventricular diastolic hemodynamics

Pulsed-wave Doppler echocardiography revealed that landlocked masu salmon and sea-run cherry salmon have different ventricular diastolic properties despite being the same age and species. In masu salmon, monophasic atrioventricular inflow waveforms were observed immediately after the P wave in the electrocardiograph (Fig 1C), and therefore, these inflows may be attributed to atrial contraction. Simultaneous measurement of atrial and ventricular pressures showed that the atrial-ventricular pressure gradient occurred during atrial systole, but not the early diastolic phase between completion of ventricular ejection and atrial systole (S4 Fig). Our results suggested that there was no ventricular suction of blood from the atrium in masu salmon. Conversely, cherry salmon mainly displayed two types of atrioventricular inflow patterns depending on the heart rate: (1) early ventricular diastolic and atrial systolic fusion and (2) biphasic waveform (Fig 1D and 1E, S4 Fig). The inflows observed before detecting the P wave indicated passive ventricular filling, suggesting that the ventricle in cherry salmon acquired the ability to suction blood from the atrium. However, the atrial systolic velocity was greater than the early diastolic velocity (Fig 1F, early diastolic velocity/atrial systolic velocity ratio = 0.48 ± 0.06); it is clear, therefore, that atrial contraction plays a dominant role in ventricular filling in cherry salmon, at least under anesthesia.

A negative correlation has been reported between body weight and resting heart rate in both mammals and birds [83, 84], but this rule does not always apply to fish [85]. However, in our study, at 29–30 mpf, cherry salmon was over 10 times heavier than masu salmon (Table 1), and the heart rate of masu salmon was higher than that of cherry salmon (S1 Table, *p* = 0.0164). A previous hemodynamic study of rainbow trout suggested that the heart rate is associated with the appearance of monophasic or biphasic atrioventricular inflow waveforms [86]. Pulsed-wave Doppler echocardiography of rainbow trout recorded biphasic waveforms in specimens with a low heart rate (44–69 bpm) [86]. In cherry salmon, monophasic atrioventricular inflow switched to biphasic when the R-R interval times extended, but the time duration between the end of ventricular ejection and the onset of new inflow was constant at 92−96 ms, independent of the R-R interval (S4 Fig). In comparison, the lower-heart-rate cherry salmon had longer ventricular diastole, and ventricular filling occurred before atrial contraction began. No biphasic inflow waveform was observed, in masu salmon, even though the time duration from ventricular ejection to the onset of atrioventricular inflow increased. Further, since juvenile Atlantic salmon of 15 g body weight showed biphasic waveforms [87], it is possible that our data derived from pulsed-wave Doppler echocardiography are species specific. Elastic reaction forces, muscle contraction, and differences in chamber size between the atria and ventricles have been considered as candidates for the driving forces of ventricular filling, but are not yet sufficiently understood [88, 89]. Our study revealed that the same species adapted to different environments show different ventricular filling systems during diastole. Thus, histological and material changes in the ventricles of cherry salmon contribute to generating the early diastolic ventricular inflow (Figs 2 and 3).

### Relative ventricular stiffness indices

The mechanical properties of ventricles of different sizes cannot simply be evaluated by comparing the individual mechanical parameters [4]. To assess ventricular contraction, end-systolic pressure–volume analysis has been carried out by normalizing ventricular volume per myocardial mass with respect to its contraction indexes [90]. EDPVR analysis, based on a time-varying elastance model, is an important method to assess ventricular stiffness [4]; however, its application in comparing the stiffness of ventricles of different sizes is difficult. In a smaller ventricle, the ratio of the pressure increase relative to the volume increase is greater than for a larger one. Thus, the EDPVR curve for a smaller heart would be steeper than that for a larger ventricle, even with the same actual stiffness. To evaluate the relative ventricular stiffness of masu and cherry salmon, we calculated the stiffness per unit of myocardial mass by normalizing the ventricular end-diastolic volume by the ventricular mass [15]. The index of ventricular stiffness per unit myocardial mass of cherry salmon, calculated from the normalized EDPVR curve (Fig 2A), was significantly higher than that of masu salmon (Fig 2B), thus suggesting that the ventricle of cherry salmon has a relatively reduced ventricular diastolic compliance.

The ventricular walls of the cherry salmon were thicker, and their coronary vascular network was well developed compared to those of the masu salmon (Fig 3D and 3H). Coronary circulation presents hemodynamic characteristics that are not observed in other organs: during ventricular systole, arterial blood flow is blocked because of the pressure associated with contraction, while during diastole, blood flows only after the percutaneous pressure is reduced. Therefore, excessive stretching of the coronary vessels associated with ventricular dilation increases vascular resistance and decreases blood supply to myocardium. Thus, the stiffening of the ventricle in cherry salmon might support the coronary vessel shapes and set the upper threshold for stroke volume.

### Collagen accumulation in the thick compact layer of cherry salmon

In this study, histological analysis provided the causes of ventricular stiffness. The ventricle of cherry salmon had a thicker compact layer compared to that of masu salmon (Fig 3D, 3H and 3K). This is consistent with previous reports on other salmonids wherein the compact layer thickens with growth [56, 57]. This histological change indicates that the growth ratio of the compact and sponge layers was different during the growth process of the masu and cherry salmon. Hypertrophy of the ventricular wall in response to hypertrophic cardiomyopathy is one of the factors impairing the diastolic function of ventricles [91, 92]. Importantly, hypertrophy of the ventricular tissue is sometimes accompanied by an increase in cardiomyocyte size [93]. Mammalian cardiomyocytes become larger and maturate via multinucleation and polyploidization after birth [94, 95]. However, there were no differences in the morphology and nuclear number of each isolated cardiomyocyte between the masu and cherry salmon (Fig 4; S7 and S8 Figs). Therefore, *O*. *masou* hearts may show growth because of proliferation of cardiomyocytes or the differentiation of progenitor and stem cells instead of cardiomyocyte hypertrophy.

The compact layer of cherry salmon also contained more collagen fibers as compared with masu salmon (Fig 3E, 3I and 3M). Increased stiffness in the ventricle correlates with tissue fibrosis [66, 96], and is reportedly induced by collagen accumulation in the myocardium [97]. In a study involving rainbow trout, breeding at lower temperatures reduced ventricular compliance mediated by accumulating collagen fibers, but not because of hypertrophy of the compact layer [65]. Hence, the stiffening of the ventricle in cherry salmon is assumed to be strongly correlated to increasing collagen fiber content in the compact layer.

The bulbus arteriosus of teleost fishes contain abundant extracellular matrixes and smooth muscle cells [98–100]. Bulbus arteriosus of masu and cherry salmon appear to contain elastin, an elastic protein, stained purple-black by Elastica van Gieson staining (Fig 3C and 3G). They also had collagen fiber (S10F–S10H Fig). The superior extensibility of the vessel provided by these extracellular matrices contributes to maintaining the blood pressure of blood ejected from the ventricles [98–100].

### Similar expression patterns of Connectin isoforms

The expression ratios of the Connectin isoforms affect cell passive tension [30, 35–37]. The mammalian heart expresses the lower-molecular-weight Connectin isoform N2B and the higher-molecular-weight Connectin isoform N2BA [31–33]. Rainbow trout express N2B-like and N2BA-like proteins in their hearts [101]. Multiple *connectin* splicing variants are reportedly expressed in zebrafish [102]. SDS-agarose gel electrophoresis showed the expression of two types of Connectins in the ventricles of the masu and cherry salmon (Fig 5A and 5B). Western blotting could not, however, discriminate between these Connectins because either of the isoforms might not have been detected by the antibody (S10 Fig). The similarity in the expression pattern of Connectins in the ventricles of cherry and masu salmon indicated that the difference in ventricular stiffness was not because of regulation by splicing of connectin molecules. There was no difference in sarcomere length under unloaded conditions as assessed by TEM (Fig 5C–5G). However, the correlation between the molecular sizes of Connectins and sarcomere length is controversial [103, 104]. The regulation of tensile strength by Connectin phosphorylation is a possible mechanism, other than the expression of its isoforms [27], but we did not analyze it.

We used *O*. *masou* of the same age but did not consider sex because it could not be specified at the time of purchase. Differences between male and female wild-type zebrafish strains at 9 mpf have been reported [105]. Furthermore, the amount of connective tissues in the ventricles of rainbow trout tends to differ based on their sex [106]. We used non-strained *O*. *masou* with non-uniform genetic backgrounds in this study, but a rigorous experimental system with a molecular basis is needed to provide definite answers to questions regarding the processes of heart development, morphogenesis, functions, and gene expression patterns.

## Conclusion

We analyzed the properties of ventricular diastole and heart histology in landlocked masu and sea-run cherry salmon at 29–30 mpf. Because cherry salmon had thick ventricular walls and accumulated collagen in the myocardium, their ventricles were found to be stiffer than those of masu salmon on a per unit myocardial mass basis. Histological adaptations of the heart because of body growth and the living environment cause hemodynamic changes in *O*. *masou* due to changes in the stiffness of the myocardium and ventricular filling system.

## Supporting information

S1 Fig Anatomical observations of a fish heart. (A) An image of the left lateral view of a masu salmon heart at 29 months post fertilization (mpf). Scale bar = 1 cm. (B) Magnified view of the white box in panel (A). At, atrium (green dashed line); V, ventricle (magenta dashed line); Ba, bulbus arteriosus (yellow dashed line); Sv, sinus venosus (white dashed line); Di, diaphragms (blue dashed line); Li, liver. (C) Life history of *Oncorhynchus masou masou*. (D) A juvenile *O*. *masou* at 6 mpf. Scale bar = 1 cm. (E) Stereomicroscopic images of a juvenile heart. Scale bar = 1 mm.
(TIF)

S2 Fig Simultaneous echocardiographic and electrocardiographic measurements of *Oncorhynchus masou masou*. (A) Method used for the fish echocardiography and electrocardiography. To record the cardiac dynamics on the sagittal axis, anesthetized fish were turned upside down and secured to the holder, and the transducer probe was vertically and directly positioned above the heart. Electrodes were clipped to the pectoral fins and pelvic fin. Air was supplied continuously during the experiments. (B, C) Echocardiographic images of the sagittal axis; (B) masu salmon at 30 months post fertilization (mpf); (C) cherry salmon at 30 mpf. At, atrium (green dashed line); V, ventricle (magenta dashed line); Ba, bulbus arteriosus (yellow dashed line); Li, liver. (D) Electrocardiography results from the body surface of the masu salmon. P: P wave, QRS: QRS complex, T: T wave.
(TIF)

S3 Fig Atrioventricular inflow waveforms at different R-R interval times. (A) Pulsed-wave Doppler images of masu (left column) and cherry salmon (right column). Upper row; representative ventricular ejection waveform (326.75 ± 1.04 ms in masu salmon #5 and 351.75 ± 1.09 ms in cherry salmon #1). Second−lower rows; atrioventricular inflow waveforms observed at short, intermediate, and long R-R interval times. The yellow line indicated the average time from the peak of the R wave to the end of ventricular ejection. The yellow double-headed arrows indicated the time from the end of ventricular ejection to the onset of atrioventricular inflow. (B) Times of R-R interval and from the end of ventricular ejection until atrioventricular inflow were observed in each panel in (A).
(TIF)

S4 Fig *In vivo* atrial-ventricular pressure analysis of masu salmon. (A, B) Recordings of the ventricular pressure in the masu salmon at 34 mpf. Ventricular and atrial pressures during three heart cycles (A). Ventricular and atrial pressure in the ventricular diastole (B); magnification of the 1 and 2 s range of panel (A). The solid and dotted lines indicate the ventricular pressure and atrial pressure, respectively. VD, ventricular diastole; IR, isovolumic relaxation; AS, atrial systole.
(TIF)

S5 Fig End-diastolic pressure–volume relationship analysis for masu and cherry salmon. (A) The horizontal axis shows the ventricular chamber volume normalized by the mass of the masu salmon ventricles (N = 4) and the cherry salmon ventricles (N = 3). (B) Ventricular mass and index of ventricular stiffness in the masu and cherry salmon. The results of the EDPVR (A) were applied to Eq (1) to obtain the exponential *C*, ○: masu salmon, ●: cherry salmon. Actual measurements are shown in the S2 File.
(TIF)

S6 Fig Hematoxylin and eosin staining of isolated fish hearts. (A–H) Hematoxylin and eosin staining images of the sagittal sections of the hearts at 29 months post fertilization: masu salmon (A–D) and cherry salmon (E–H). (A, E) Images of the whole heart. Scale bars = 5 mm. (B, F) Magnified images of the ventricles. Scale bars = 1 mm. (C, G) High-magnification images of the compact layers. Scale bars = 100 μm. (D, H) High-magnification images of the spongy layers. Scale bars = 100 μm. The cytoplasm is shown in magenta, and the nuclei are shown in blue–purple. At, atrium; V, ventricle; Ba, bulbus arteriosus; Cl, compact layer; Sl, spongy layer.
(TIF)

S7 Fig Immunofluorescence staining of cardiomyocytes. (A–F) Immunofluorescence images show attached cardiomyocytes from the masu and cherry salmon at 29 months post fertilization. Cardiomyocytes were stained with antibodies recognizing Alpha-actinin-2 at z-lines (green) and Hoechst 33342 to detect nuclei (blue). (B, D) Magnifications of the images in (A) and (C). (E, F) Merged bright-field and immunostaining images. (F) Non- cardiomyocyte; magnification view of the white dotted line enclosure in (E). Scale bars = 20 μm in (A), (C) and (E), and 5 μm in (B), (D) and (F).
(TIF)

S8 Fig A binucleated cardiomyocyte. (A) A Hoechst 33342-stained binucleated cardiomyocyte (blue) in a cherry salmon. (B) Magnified image shows Hoechst 33342 signals in the grayscale mode. Scale bars = 20 μm.
(TIF)

S9 Fig Anti-Connectin antibody immunoblots. Representative and original immunoblot image showing the detection of Connectin expression in masu (Ma) and cherry (Ch) salmon hearts. Connectin N2A isoform in the mouse skeletal muscle in lanes 1 and 9 (M1, 3,700 kDa) and the N2B isoform in the left ventricle of mice in lanes 2 and 10 (M2, 3,000 kDa) were used as a positive control for the experiment and as a guide for molecular weight. α-connectin (T1) and ß-connectin (T2) indicate intact Connectins and their degraded products, respectively. A raw data image of western blot is found in S1 Raw images.
(TIF)

S10 Fig Electron micrographs of masu and cherry salmon hearts. (A–D) Representative transmission electron microscopy images of the cross-sections and longitudinal sections of the atrial sarcomeres of masu and cherry salmon at 29 months post fertilization (mpf). Insets of (A, C) showed higher magnifications of cross-sections of the myofibril bundles. (E) The graph showed atrium sarcomere lengths of the z-line to z-line in masu salmon (2.13 ± 0.16 μm, N = 25) and cherry salmon (2.12 ± 0.11 μm, N = 103). One or two ultrathin slices were observed in each tissue sample. Lines and error bars indicate means ± standard deviations. ○: masu salmon, ●: cherry salmon. (F–H) Representative transmission electron microscopy images of the bulbus arteriosus of masu and cherry salmon at 29 mpf. (G) Higher magnification image in the black dashed line in (F). FM: microfilament; CF: collagen fiber Scale bars = 500 nm in (A, C), 1 μm in (B, D), and 2 μm in (F–H). Minimal data sets for (E) are found in S5 File.
(TIF)

S1 Table Heart rates of the masu and cherry salmon. (DOCX)

S1 Movie Longitudinal axis echocardiography of a masu salmon. (MP4)

S2 Movie Longitudinal axis echocardiography of a cherry salmon. (MP4)

S1 File Minimal data sets for atrioventricular velocities. This file is listed the peak velocities in the masu salmon atrial systolic waveforms, the early diastolic/atrial systolic fused waveforms in cherry salmon, the cherry salmon early diastolic waveforms, and the cherry salmon atrial systolic waveforms. These were calculated from the pulse wave Doppler image.
(XLSX)

S2 File Minimal data sets for EDPVR analysis. (XLSX)

S3 File Minimal data sets of Fig 3K–3M. This file contains values of compact layer area, total ventricular area on sagittal heart slices, the number of nuclei in each image, total cell area in each image, collagen fiber area in each image.
(XLSX)

S4 File Minimal data sets for the morphology analysis of the isolated cardiomyocytes. This file contains values for the long axis length and area of the isolated cardiomyocytes in masu and cherry salmon.
(XLSX)

S5 File Minimal data sets for sarcomere lengths. This file contains values for sarcomere length of atrium and ventricle in masu and cherry salmon, respectively.
(XLSX)

S1 Raw images Raw data images for Fig 5A and S9 Fig. (PDF)

## References

[1] Nagueh SF. Left Ventricular Diastolic Function: Understanding Pathophysiology, Diagnosis, and Prognosis With Echocardiography. JACC Cardiovasc Imaging. 2020;13(1 Pt 2):228–44. doi: 10.1016/j.jcmg.2018.10.038 30982669

[2] Salmasi AM, Alimo A, Jepson E, Dancy M. Age-associated changes in left ventricular diastolic function are related to increasing left ventricular mass. Am J Hypertens. 2003;16(6):473–7. doi: 10.1016/s0895-7061(03)00846-x . 12799096

[3] Lee SL, Daimon M, Di Tullio MR, Homma S, Nakao T, Kawata T, et al. Relationship of Left Ventricular Diastolic Function to Obesity and Overweight in a Japanese Population With Preserved Left Ventricular Ejection Fraction. Circ J. 2016;80(9):1951–6. doi: 10.1253/circj.CJ-16-0381 . 27385498

[4] Burkhoff D, Mirsky I, Suga H. Assessment of systolic and diastolic ventricular properties via pressure-volume analysis: a guide for clinical, translational, and basic researchers. Am J Physiol Heart Circ Physiol. 2005;289(2):H501–12. doi: 10.1152/ajpheart.00138.2005 . 16014610

[5] Appleton CP, Hatle LK, Popp RL. Relation of transmitral flow velocity patterns to left ventricular diastolic function: new insights from a combined hemodynamic and Doppler echocardiographic study. J Am Coll Cardiol. 1988;12(2):426–40. doi: 10.1016/0735-1097(88)90416-0 . 3392336

[6] Starling EH. The Linacre Lecture on the Law of the Heart, Given at Cambridge, 1915. Nature. 1918;101(2525):43–43. 10.1038/101043a0

[7] Sequeira V, van der Velden J. Historical perspective on heart function: the Frank-Starling Law. Biophys Rev. 2015;7(4):421–47. doi: 10.1007/s12551-015-0184-4 . 28510104 PMC5418489

[8] Frank O. Zur Dynamik des Herzmuskels. Z Biol. 1895;32:370–447. https://ci.nii.ac.jp/naid/10008300348/en/

[9] Burch GE, Cronvich JA, Creech O, Hyman A. Pressure-volume diagrams of the left ventricle of man; a preliminary report. Am Heart J. 1957;53(6):890–4. doi: 10.1016/0002-8703(57)90325-3 . 13424469

[10] Hiroyuki S. Time Course of Left Ventricular Pressure-Volume Relationship under Various Enddiastolic Volume. Japanese Heart Journal. 1969;10(6):509–15. doi: 10.1536/ihj.10.509 . 5308142

[11] Suga H, Sagawa K. Instantaneous pressure-volume relationships and their ratio in the excised, supported canine left ventricle. Circ Res. 1974;35(1):117–26. doi: 10.1161/01.res.35.1.117 . 4841253

[12] Honda T, Ujihara Y, Hanashima A, Hashimoto K, Tanemoto K, Mohri S. Turtle spongious ventricles exhibit more compliant diastolic property and possess larger elastic regions of connectin in comparison to rat compact left ventricles. Kawasaki Medical Journal. 2018;44(1):1–17. 10.11482/KMJ-E44(1)1

[13] Peterson KL, Tsuji J, Johnson A, DiDonna J, LeWinter M. Diastolic left ventricular pressure-volume and stress-strain relations in patients with valvular aortic stenosis and left ventricular hypertrophy. Circulation. 1978;58(1):77–89. doi: 10.1161/01.cir.58.1.77 . 148335

[14] Mirsky I, Pasipoularides A. Elastic properties of normal and hypertrophied cardiac muscle. Fed Proc. 1980;39(2):156–61. https://www.ncbi.nlm.nih.gov/pubmed/6444389 . 6444389

[15] Mitsuyama S, Takeshita D, Obata K, Zhang GX, Takaki M. Left ventricular mechanical and energetic changes in long-term isoproterenol-induced hypertrophied hearts of SERCA2a transgenic rats. J Mol Cell Cardiol. 2013;59:95–106. doi: 10.1016/j.yjmcc.2013.02.012 . 23458361

[16] Klotz S, Hay I, Dickstein ML, Yi GH, Wang J, Maurer MS, et al. Single-beat estimation of end-diastolic pressure-volume relationship: a novel method with potential for noninvasive application. Am J Physiol Heart Circ Physiol. 2006;291(1):H403–12. doi: 10.1152/ajpheart.01240.2005 . 16428349

[17] Maruyama K. Connectin, an elastic protein from myofibrils. J Biochem. 1976;80(2):405–7. doi: 10.1093/oxfordjournals.jbchem.a131291 . 1002676

[18] Maruyama K, Kimura S, Kuroda M, Handa S. Connectin, an elastic protein of muscle. Its abundance in cardiac myofibrils. J Biochem. 1977;82(2):347–50. https://www.ncbi.nlm.nih.gov/pubmed/914785 . 914785

[19] Maruyama K, Natori R, Nonomura Y. New elastic protein from muscle. Nature. 1976;262(5563):58–60. doi: 10.1038/262058a0 . 934326

[20] Wang K, McClure J, Tu A. Titin: major myofibrillar components of striated muscle. Proc Natl Acad Sci U S A. 1979;76(8):3698–702. doi: 10.1073/pnas.76.8.3698 . 291034 PMC383900

[21] Linke WA, Popov VI, Pollack GH. Passive and active tension in single cardiac myofibrils. Biophys J. 1994;67(2):782–92. doi: 10.1016/S0006-3495(94)80538-7 . 7948691 PMC1225421

[22] Granzier HL, Wang K. Interplay between passive tension and strong and weak binding cross-bridges in insect indirect flight muscle. A functional dissection by gelsolin-mediated thin filament removal. J Gen Physiol. 1993;101(2):235–70. doi: 10.1085/jgp.101.2.235 . 7681097 PMC2216761

[23] Granzier HL, Irving TC. Passive tension in cardiac muscle: contribution of collagen, titin, microtubules, and intermediate filaments. Biophys J. 1995;68(3):1027–44. doi: 10.1016/S0006-3495(95)80278-X . 7756523 PMC1281826

[24] Labeit S, Kolmerer B. Titins: giant proteins in charge of muscle ultrastructure and elasticity. Science. 1995;270(5234):293–6. doi: 10.1126/science.270.5234.293 . 7569978

[25] Bang ML, Centner T, Fornoff F, Geach AJ, Gotthardt M, McNabb M, et al. The complete gene sequence of titin, expression of an unusual approximately 700-kDa titin isoform, and its interaction with obscurin identify a novel Z-line to I-band linking system. Circ Res. 2001;89(11):1065–72. doi: 10.1161/hh2301.100981 . 11717165

[26] Watanabe K, Nair P, Labeit D, Kellermayer MS, Greaser M, Labeit S, et al. Molecular mechanics of cardiac titin’s PEVK and N2B spring elements. J Biol Chem. 2002;277(13):11549–58. doi: 10.1074/jbc.M200356200 . 11799131

[27] Yamasaki R, Wu Y, McNabb M, Greaser M, Labeit S, Granzier H. Protein kinase A phosphorylates titin’s cardiac-specific N2B domain and reduces passive tension in rat cardiac myocytes. Circ Res. 2002;90(11):1181–8. doi: 10.1161/01.res.0000021115.24712.99 . 12065321

[28] LeWinter MM, Granzier HL. Titin Is a Major Human Disease Gene. Circulation. 2013;127(8):938–44. doi: 10.1161/CIRCULATIONAHA.112.139717 . 23439446 PMC3594684

[29] Linke WA, Hamdani N. Gigantic business: titin properties and function through thick and thin. Circ Res. 2014;114(6):1052–68. doi: 10.1161/CIRCRESAHA.114.301286 . 24625729

[30] Freiburg A, Trombitas K, Hell W, Cazorla O, Fougerousse F, Centner T, et al. Series of exon-skipping events in the elastic spring region of titin as the structural basis for myofibrillar elastic diversity. Circ Res. 2000;86(11):1114–21. doi: 10.1161/01.res.86.11.1114 . 10850961

[31] Opitz CA, Leake MC, Makarenko I, Benes V, Linke WA. Developmentally regulated switching of titin size alters myofibrillar stiffness in the perinatal heart. Circ Res. 2004;94(7):967–75. doi: 10.1161/01.RES.0000124301.48193.E1 . 14988228

[32] Lahmers S, Wu Y, Call DR, Labeit S, Granzier H. Developmental control of titin isoform expression and passive stiffness in fetal and neonatal myocardium. Circ Res. 2004;94(4):505–13. doi: 10.1161/01.RES.0000115522.52554.86 . 14707027

[33] Greaser ML, Krzesinski PR, Warren CM, Kirkpatrick B, Campbell KS, Moss RL. Developmental changes in rat cardiac titin/connectin: transitions in normal animals and in mutants with a delayed pattern of isoform transition. J Muscle Res Cell Motil. 2005;26(6–8):325–32. doi: 10.1007/s10974-005-9039-0 . 16491431

[34] Warren CM, Krzesinski PR, Campbell KS, Moss RL, Greaser ML. Titin isoform changes in rat myocardium during development. Mech Dev. 2004;121(11):1301–12. doi: 10.1016/j.mod.2004.07.003 . 15454261

[35] Trombitas K, Wu Y, Labeit D, Labeit S, Granzier H. Cardiac titin isoforms are coexpressed in the half-sarcomere and extend independently. Am J Physiol Heart Circ Physiol. 2001;281(4):H1793–9. doi: 10.1152/ajpheart.2001.281.4.H1793 . 11557573

[36] LeWinter MM, Granzier H. Cardiac titin: a multifunctional giant. Circulation. 2010;121(19):2137–45. doi: 10.1161/CIRCULATIONAHA.109.860171 . 20479164 PMC2905226

[37] Cazorla O, Freiburg A, Helmes M, Centner T, McNabb M, Wu Y, et al. Differential expression of cardiac titin isoforms and modulation of cellular stiffness. Circ Res. 2000;86(1):59–67. doi: 10.1161/01.res.86.1.59 . 10625306

[38] Nawar G. On the anatomy of clarias lazera. Journal of Morphology. 1954;94(3):551–85. 10.1002/jmor.1050940304

[39] Saxena DB, Bakhshi PL. Cardio-Vascular System of Some Fishes of the Torrential Streams in India. Part I. Heart of Orienus plagiostomus plagiostomus and Botia birdi. Japanese Journal of Ichthyology. 1965;12(3–6):70–81. 10.11369/jji1950.12.70

[40] Randall DJ. Functional morphology of the heart in fishes. Am Zool. 1968;8(2):179–89. doi: 10.1093/icb/8.2.179 . 5738636

[41] Farrell AP, Jones DR. 1—The Heart. In: Hoar WS, Randall DJ, Farrell AP, editors. Fish Physiology. 12: Academic Press; 1992. p. 1–88.

[42] Pieperhoff S, Bennett W, Farrell AP. The intercellular organization of the two muscular systems in the adult salmonid heart, the compact and the spongy myocardium. J Anat. 2009;215(5):536–47. doi: 10.1111/j.1469-7580.2009.01129.x . 19627390 PMC2780571

[43] Davie PS, Farrell AP. The coronary and luminal circulations of the myocardium of fishes. Canadian Journal of Zoology. 1991;69(7):1993–2001. 10.1139/z91-278

[44] Tota B. Vascular and metabolic zonation in the ventricular myocardium of mammals and fishes. Comp Biochem Physiol A Comp Physiol. 1983;76(3):423–37. doi: 10.1016/0300-9629(83)90442-5 . 6139219

[45] Tota B, Cimini V, Salvatore G, Zummo G. Comparative study of the arterial and lacunary systems of the ventricular myocardium of elasmobranch and teleost fishes. Am J Anat. 1983;167(1):15–32. doi: 10.1002/aja.1001670103 . 6869307

[46] Myoarchitecture Tota B. and vascularization of the elasmobranch heart ventricle. Journal of Experimental Zoology. 1989;252(S2):122–35. 10.1002/jez.1402520413

[47] Nelson J, Grande T, Wilson M. Fishes of the World. 5 ed. 2016.

[48] Sherrill J, Weber ES 3rd, Marty GD, Hernandez-Divers S. Fish cardiovascular physiology and disease. Vet Clin North Am Exot Anim Pract. 2009;12(1):11–38. doi: 10.1016/j.cvex.2008.08.002 . 19131028

[49] Sandblom E, Axelsson M. Venous hemodynamic responses to acute temperature increase in the rainbow trout (Oncorhynchus mykiss). Am J Physiol Regul Integr Comp Physiol. 2007;292(6):R2292–8. doi: 10.1152/ajpregu.00884.2006 . 17322113

[50] Farrell AP. Cardiorespiratory performance in salmonids during exercise at high temperature: insights into cardiovascular design limitations in fishes. Comp Biochem Physiol A Mol Integr Physiol. 2002;132(4):797–810. doi: 10.1016/s1095-6433(02)00049-1 . 12095864

[51] Currie S, Evans DH. The Physiology of Fishes. 5 th ed. Boca Raton: CRC Press; 2020.

[52] Claireaux G, McKenzie DJ, Genge AG, Chatelier A, Aubin J, Farrell AP. Linking swimming performance, cardiac pumping ability and cardiac anatomy in rainbow trout. J Exp Biol. 2005;208(Pt 10):1775–84. doi: 10.1242/jeb.01587 . 15879059

[53] Poppe TT, Johansen R, Gunnes G, Torud B. Heart morphology in wild and farmed Atlantic salmon Salmo salar and rainbow trout Oncorhynchus mykiss. Dis Aquat Organ. 2003;57(1–2):103–8. doi: 10.3354/dao057103 . 14735927

[54] Agnisola C, Tota B. Structure and function of the fish cardiac ventricle: flexibility and limitations. Cardioscience. 1994;5(3):145–53. https://www.ncbi.nlm.nih.gov/pubmed/7827250 . 7827250

[55] Tota B, Gattuso A. Heart ventricle pumps in teleosts and elasmobranchs: A morphodynamic approach. Journal of Experimental Zoology. 1996;275(2–3):162–71. 10.1002/(SICI)1097-010X(19960601/15)275:2/3<162::AID-JEZ8>3.0.CO;2-B

[56] Poupa O, Gesser H, Jonsson S, Sullivan L. Coronary-supplied compact shell of ventricular myocardium in salmonids: growth and enzyme pattern. Comp Biochem Physiol A Comp Physiol. 1974;48(1):85–95. doi: 10.1016/0300-9629(74)90856-1 . 4151635

[57] Farrell AP, Hammons AM, Graham MS, Tibbits GF. Cardiac growth in rainbow trout, Salmo gairdneri. Canadian Journal of Zoology. 1988;66(11):2368–73. 10.1139/z88-351

[58] Johansen IB, Lunde IG, Røsjø H, Christensen G, Nilsson GE, Bakken M, et al. Cortisol response to stress is associated with myocardial remodeling in salmonid fishes. J Exp Biol. 2011;214(Pt 8):1313–21. doi: 10.1242/jeb.053058 . 21430209

[59] Grethe R, Helge S, Sigurd E, FraserDylan. Offspring size effects vary over fine spatio-temporal scales in Atlantic salmon (Salmo salar). Canadian Journal of Fisheries and Aquatic Sciences. 2013;70(1):5–12. 10.1139/cjfas-2012-0152

[60] Franklin CE, Davie PS. Sexual Maturity Can Double Heart Mass and Cardiac Power Output in Male Rainbow Trout. Journal of Experimental Biology. 1992;171(1):139–48. 10.1242/jeb.171.1.139

[61] Clark RJ, Rodnick KJ. Morphometric and biochemical characteristics of ventricular hypertrophy in male rainbow trout (Oncorhynchus mykiss). J Exp Biol. 1998;201(Pt 10):1541–52. https://www.ncbi.nlm.nih.gov/pubmed/9556537 doi: 10.1242/jeb.201.10.1541 9556537

[62] Brijs J, Sandblom E, Dekens E, Näslund J, Ekström A, Axelsson M. Cardiac remodeling and increased central venous pressure underlie elevated stroke volume and cardiac output of seawater-acclimated rainbow trout. American Journal of Physiology-Regulatory, Integrative and Comparative Physiology. 2017;312(1):R31–R39. doi: 10.1152/ajpregu.00374.2016 . 27903511

[63] Farrell AP, Johansen JA, Suarez RK. Effects of exercise-training on cardiac performance and muscle enzymes in rainbow trout,Oncorhynchus mykiss. Fish Physiol Biochem. 1991;9(4):303–12. doi: 10.1007/BF02265151 . 24213727

[64] Norstrud KS, Vindas MA, Nilsson GE, Johansen IB. Short-term cortisol exposure alters cardiac hypertrophic and non-hypertrophic signalling in a time-dependent manner in rainbow trout. Biol Open. 2018;7(12) doi: 10.1242/bio.037853 . 30341103 PMC6310887

[65] Keen AN, Fenna AJ, McConnell JC, Sherratt MJ, Gardner P, Shiels HA. The Dynamic Nature of Hypertrophic and Fibrotic Remodeling of the Fish Ventricle. Front Physiol. 2015;6:427. doi: 10.3389/fphys.2015.00427 . 26834645 PMC4720793

[66] Fomovsky GM, Thomopoulos S, Holmes JW. Contribution of extracellular matrix to the mechanical properties of the heart. J Mol Cell Cardiol. 2010;48(3):490–6. doi: 10.1016/j.yjmcc.2009.08.003 . 19686759 PMC2823835

[67] Kato F. Life histories of masu and amago salmon (Oncorhynchus masou and Oncorhynchus rhodurus. Pacific Salmon Life Histories. 1991:446–520. https://ci.nii.ac.jp/naid/10012483661/en/

[68] McPhail JD. The Origin and Speciation of Oncorhynchus Revisited. In: Stouder DJ, Bisson PA, Naiman RJ, editors. Pacific Salmon & their Ecosystems: Status and Future Options. Boston, MA: Springer US; 1997. p. 29–38.

[69] Kazuhiro U, Akihiko H, Kohei Y. Serum thyroid hormone, guanine and protein profiles during smoltification and after thyroxine treatment in the masu salmon, Oncorhynchus masou. Comparative Biochemistry and Physiology Part A: Physiology. 1994;107(4):607–12. 10.1016/0300-9629(94)90359-X

[70] Carruth LL, Jones RE, Norris DO. Cortisol and Pacific Salmon: A New Look at the Role of Stress Hormones in Olfaction and Home-stream Migration. Integrative and Comparative Biology. 2002;42(3):574–81. doi: 10.1093/icb/42.3.574 . 21708753

[71] Munakata A. Effects of growth hormone and cortisol on the downstream migratory behavior in masu salmon, Oncorhynchus masou. Gen Comp Endocrinol. 2007;150(1):12–17. doi: 10.1016/j.ygcen.2006.07.009 . 16996063

[72] Munakata A, Amano M, Ikuta K, Kitamura S, Aida K. Involvement of sex steroids and thyroid hormones in upstream and downstream behaviors in masu salmon, Oncorhynchus masou. Aquaculture. 2012;362–363:158–66. 10.1016/j.aquaculture.2010.11.027

[73] Riley JP, Skirrow G, Chester R. Chemical Oceanography. 2 nd ed. London; New York: Academic Press; 1975.

[74] Topic Popovic N, Strunjak-Perovic I, Coz-Rakovac R, Barisic J, Jadan M, Persin Berakovic A, et al. Tricaine methane-sulfonate (MS-222) application in fish anaesthesia. Journal of Applied Ichthyology. 2012;28(4):553–64. 10.1111/j.1439-0426.2012.01950.x

[75] Schindelin J, Arganda-Carreras I, Frise E, Kaynig V, Longair M, Pietzsch T, et al. Fiji: an open-source platform for biological-image analysis. Nat Methods. 2012;9(7):676–82. doi: 10.1038/nmeth.2019 . 22743772 PMC3855844

[76] Sander V, Sune G, Jopling C, Morera C, Izpisua Belmonte JC. Isolation and in vitro culture of primary cardiomyocytes from adult zebrafish hearts. Nat Protoc. 2013;8(4):800–9. doi: 10.1038/nprot.2013.041 . 23538883

[77] Zhu C, Guo W. Detection and quantification of the giant protein titin by SDS-agarose gel electrophoresis. MethodsX. 2017;4:320–27. doi: 10.1016/j.mex.2017.09.007 . 29872636 PMC5986978

[78] Soeno Y, Yajima H, Kawamura Y, Kimura S, Maruyama K, Obinata T. Organization of connectin/titin filaments in sarcomeres of differentiating chicken skeletal muscle cells. Mol Cell Biochem. 1999;190(1–2):125–31. https://www.ncbi.nlm.nih.gov/pubmed/10098979 10098979

[79] Lien S, Koop BF, Sandve SR, Miller JR, Kent MP, Nome T, et al. The Atlantic salmon genome provides insights into rediploidization. Nature. 2016;533(7602):200–5. doi: 10.1038/nature17164 . 27088604 PMC8127823

[80] Moriyama Y, Ito F, Takeda H, Yano T, Okabe M, Kuraku S, et al. Evolution of the fish heart by sub/neofunctionalization of an elastin gene. Nat Commun. 2016;7:10397. doi: 10.1038/ncomms10397 . 26783159 PMC4735684

[81] Kruger M, Sachse C, Zimmermann WH, Eschenhagen T, Klede S, Linke WA. Thyroid hormone regulates developmental titin isoform transitions via the phosphatidylinositol-3-kinase/ AKT pathway. Circ Res. 2008;102(4):439–47. doi: 10.1161/CIRCRESAHA.107.162719 . 18096819

[82] Icardo JM. Collagen and elastin histochemistry of the teleost bulbus arteriosus: false positives. Acta Histochem. 2013;115(2):185–9. doi: 10.1016/j.acthis.2012.03.002 . 22494613

[83] Welty JC, Baptista L. The life of birds. 4 th ed. New York: Cengage Learning; 1988.

[84] Holt JP, Rhode EA, Kines H. Ventricular volumes and body weight in mammals. Am J Physiol. 1968;215(3):704–15. doi: 10.1152/ajplegacy.1968.215.3.704 . 5671010

[85] Clark TD, Farrell AP. Effects of body mass on physiological and anatomical parameters of mature salmon: evidence against a universal heart rate scaling exponent. J Exp Biol. 2011;214(Pt 6):887–93. doi: 10.1242/jeb.051607 . 21346114

[86] Cotter PA, Han AJ, Everson JJ, Rodnick KJ. Cardiac hemodynamics of the rainbow trout (Oncorhynchus mykiss) using simultaneous Doppler echocardiography and electrocardiography. J Exp Zool A Ecol Genet Physiol. 2008;309(5):243–54. doi: 10.1002/jez.453 . 18366108

[87] Muir CA, Neff BD, Damjanovski S. Adaptation of a mouse Doppler echocardiograph system for assessing cardiac function and thermal performance in a juvenile salmonid. Conserv Physiol. 2021;9(1):coab070. doi: 10.1093/conphys/coab070 . 34512992 PMC8415535

[88] Maksuti E, Carlsson M, Arheden H, Kovacs SJ, Broome M, Ugander M. Hydraulic forces contribute to left ventricular diastolic filling. Sci Rep. 2017;7:43505. doi: 10.1038/srep43505 . 28256604 PMC5334655

[89] Lunkenheimer PP. Systolic ventricular filling. Eur J Cardiothorac Surg. 2004;26(3):662–3; author reply 63–4. doi: 10.1016/j.ejcts.2004.05.024 . 15302074

[90] Suga H, Hisano R, Goto Y, Yamada O. Normalization of end-systolic pressure-volume relation and Emax of different sized hearts. Jpn Circ J. 1984;48(2):136–43. doi: 10.1253/jcj.48.136 . 6700110

[91] Wigle ED, Rakowski H, Kimball BP, Williams WG. Hypertrophic cardiomyopathy. Clinical spectrum and treatment. Circulation. 1995;92(7):1680–92. doi: 10.1161/01.cir.92.7.1680 . 7671349

[92] Maron BJ, Bonow RO, Cannon RO 3rd, Leon MB, Epstein SE. Hypertrophic cardiomyopathy. Interrelations of clinical manifestations, pathophysiology, and therapy (2). N Engl J Med. 1987;316(14):844–52. doi: 10.1056/NEJM198704023161405 . 3547135

[93] Tamura T, Onodera T, Said S, Gerdes AM. Correlation of myocyte lengthening to chamber dilation in the spontaneously hypertensive heart failure (SHHF) rat. J Mol Cell Cardiol. 1998;30(11):2175–81. doi: 10.1006/jmcc.1998.0775 . 9925355

[94] Ikenishi A, Okayama H, Iwamoto N, Yoshitome S, Tane S, Nakamura K, et al. Cell cycle regulation in mouse heart during embryonic and postnatal stages. Dev Growth Differ. 2012;54(8):731–8. doi: 10.1111/j.1440-169X.2012.01373.x . 22957921

[95] Brodsky V, Sarkisov DS, Arefyeva AM, Panova NW, Gvasava IG. Polyploidy in cardiac myocytes of normal and hypertrophic human hearts; range of values. Virchows Arch. 1994;424(4):429–35. doi: 10.1007/BF00190566 . 8205355

[96] Weber KT, Brilla CG, Janicki JS. Myocardial fibrosis: functional significance and regulatory factors. Cardiovasc Res. 1993;27(3):341–8. doi: 10.1093/cvr/27.3.341 . 8490934

[97] Villari B, Campbell SE, Hess OM, Mall G, Vassalli G, Weber KT, et al. Influence of collagen network on left ventricular systolic and diastolic function in aortic valve disease. J Am Coll Cardiol. 1993;22(5):1477–84. doi: 10.1016/0735-1097(93)90560-n . 8227808

[98] Keith DA, Paz A, Gallop PM, Glimcher MJ. Histologic and biochemical identification and characterization of an elastin in cartilage. J Histochem Cytochem. 1977;25(10):1154–62. doi: 10.1177/25.10.72098 72098

[99] Braun MH, Brill RW, Gosline JM, Jones DR. Form and function of the bulbus arteriosus in yellowfin tuna (Thunnus albacares), bigeye tuna (Thunnus obesus) and blue marlin (Makaira nigricans): static properties. J Exp Biol. 2003;206(Pt 19):3311–26. doi: 10.1242/jeb.00575 . 12939364

[100] Braun MH, Brill RW, Gosline JM, Jones DR. Form and function of the bulbus arteriosus in yellowfin tuna (Thunnus albacares): dynamic properties. J Exp Biol. 2003;206(Pt 19):3327–35. doi: 10.1242/jeb.00576 . 12939365

[101] Patrick SM, Hoskins AC, Kentish JC, White E, Shiels HA, Cazorla O. Enhanced length-dependent Ca2+ activation in fish cardiomyocytes permits a large operating range of sarcomere lengths. J Mol Cell Cardiol. 2010;48(5):917–24. doi: 10.1016/j.yjmcc.2010.02.008 . 20170661

[102] Hanashima A, Hashimoto K, Ujihara Y, Honda T, Yobimoto T, Kodama A, et al. Complete primary structure of the I-band region of connectin at which mechanical property is modulated in zebrafish heart and skeletal muscle. Gene. 2017;596:19–26. doi: 10.1016/j.gene.2016.10.010 . 27725266

[103] Greaser ML, Pleitner JM. Titin isoform size is not correlated with thin filament length in rat skeletal muscle. Front Physiol. 2014;5:35. doi: 10.3389/fphys.2014.00035 . 24550844 PMC3910082

[104] Castillo A, Nowak R, Littlefield KP, Fowler VM, Littlefield RS. A nebulin ruler does not dictate thin filament lengths. Biophys J. 2009;96(5):1856–65. doi: 10.1016/j.bpj.2008.10.053 . 19254544 PMC2717268

[105] Wang LW, Huttner IG, Santiago CF, Kesteven SH, Yu ZY, Feneley MP, et al. Standardized echocardiographic assessment of cardiac function in normal adult zebrafish and heart disease models. Dis Model Mech. 2017;10(1):63–76. doi: 10.1242/dmm.026989 . 28067629 PMC5278526

[106] Klaiman JM, Fenna AJ, Shiels HA, Macri J, Gillis TE. Cardiac remodeling in fish: strategies to maintain heart function during temperature Change. PLoS One. 2011;6(9):e24464. doi: 10.1371/journal.pone.0024464 . 21915331 PMC3168507

