# Supplementary figures and images for: Cardiac hemodynamics and ventricular stiffness of sea-run cherry salmon (Oncorhynchus masou masou) differ critically from those of landlocked masu salmon
Source: PLoS One. 2022 Nov 4;17(11):e0267264. doi: 10.1371/journal.pone.0267264 (PMC9635730; doi:10.1371/journal.pone.0267264)

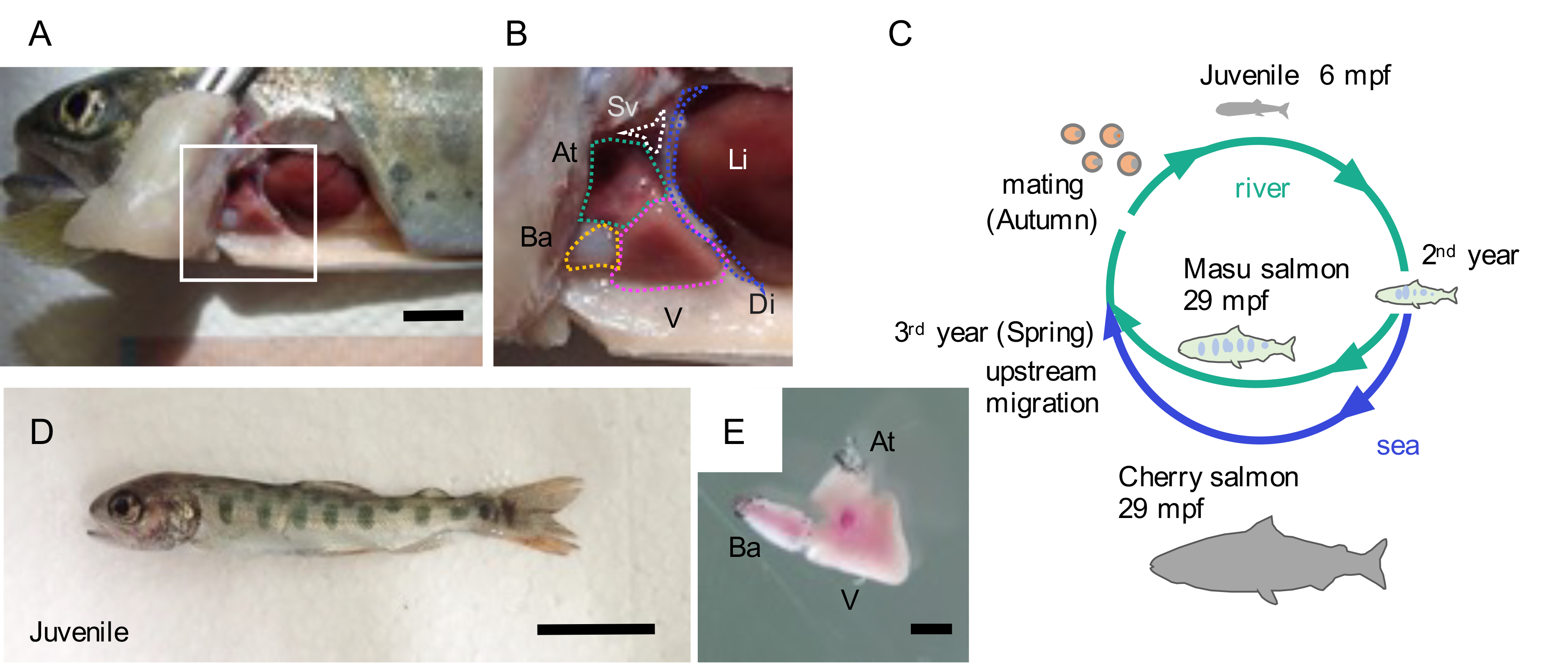

Supplement: S1 Fig — (A) An image of the left lateral view of a masu salmon heart at 29 months post fertilization (mpf). Scale bar = 1 cm. (B) Magnified view of the white box in panel (A). At, atrium (green dashed line); V, ventricle (magenta dashed line); Ba, bulbus arteriosus (yellow dashed line); Sv, sinus venosus (white dashed line); Di, diaphragms (blue dashed line); Li, liver. (C) Life history of Oncorhynchus masou masou. (D) A juvenile O. masou at 6 mpf. Scale bar = 1 cm. (E) Stereomicroscopic images of a juvenile heart. Scale bar = 1 mm. (TIF) [file pone.0267264.s001.tif]

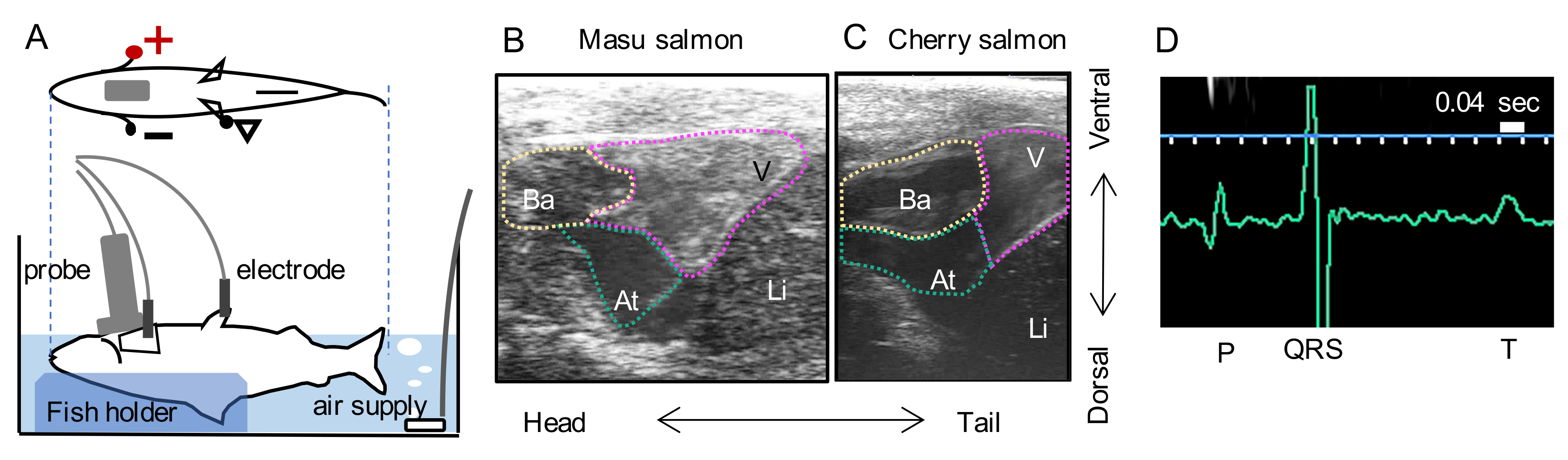

Supplement: S2 Fig — (A) Method used for the fish echocardiography and electrocardiography. To record the cardiac dynamics on the sagittal axis, anesthetized fish were turned upside down and secured to the holder, and the transducer probe was vertically and directly positioned above the heart. Electrodes were clipped to the pectoral fins and pelvic fin. Air was supplied continuously during the experiments. (B, C) Echocardiographic images of the sagittal axis; (B) masu salmon at 30 months post fertilization (mpf); (C) cherry salmon at 30 mpf. At, atrium (green dashed line); V, ventricle (magenta dashed line); Ba, bulbus arteriosus (yellow dashed line); Li, liver. (D) Electrocardiography results from the body surface of the masu salmon. P: P wave, QRS: QRS complex, T: T wave. (TIF) [file pone.0267264.s002.tif]

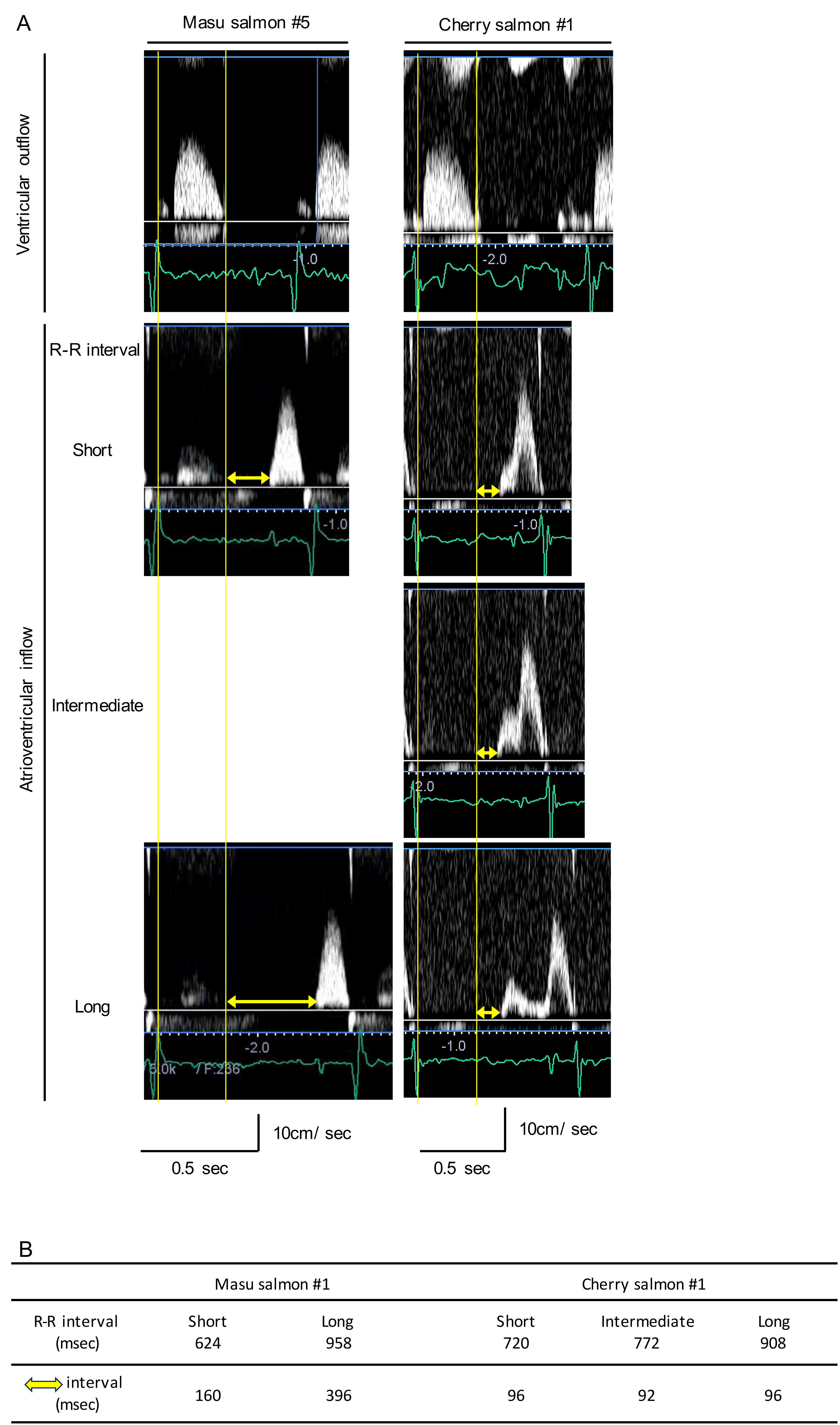

Supplement: S3 Fig — (A) Pulsed-wave Doppler images of masu (left column) and cherry salmon (right column). Upper row; representative ventricular ejection waveform (326.75 ± 1.04 ms in masu salmon #5 and 351.75 ± 1.09 ms in cherry salmon #1). Second−lower rows; atrioventricular inflow waveforms observed at short, intermediate, and long R-R interval times. The yellow line indicated the average time from the peak of the R wave to the end of ventricular ejection. The yellow double-headed arrows indicated the time from the end of ventricular ejection to the onset of atrioventricular inflow. (B) Times of R-R interval and from the end of ventricular ejection until atrioventricular inflow were observed in each panel in (A). (TIF) [file pone.0267264.s003.tif]

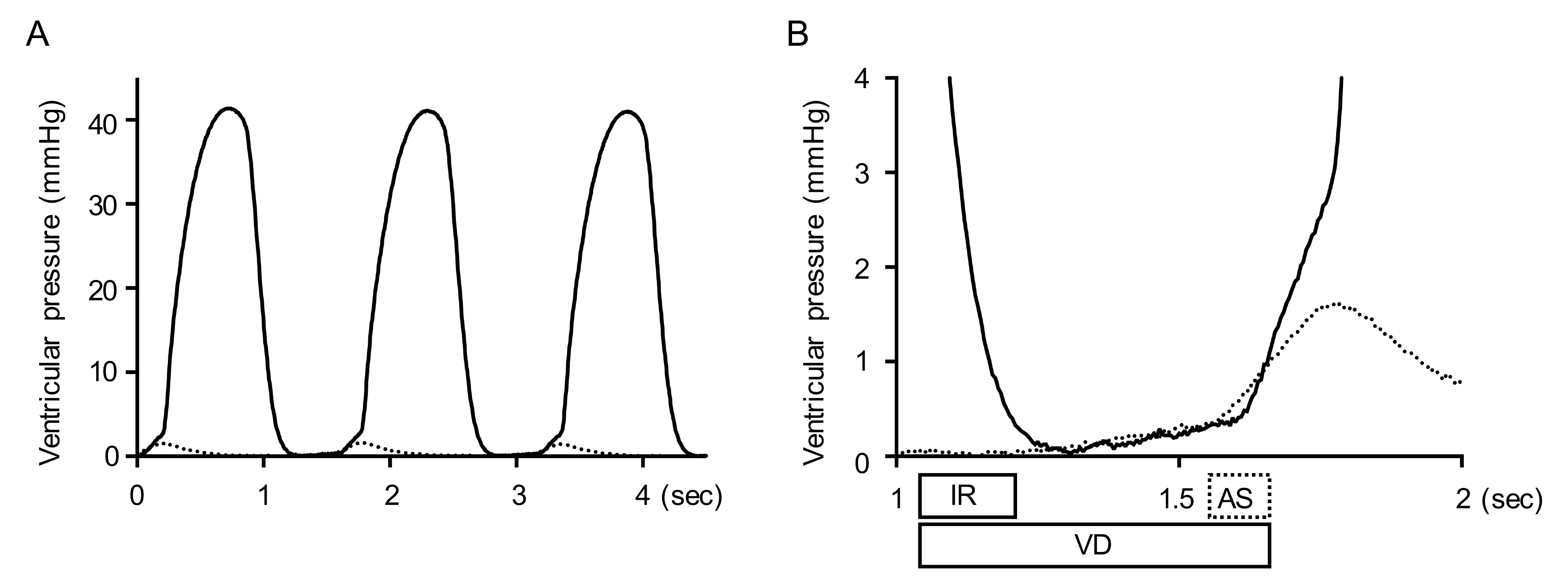

Supplement: S4 Fig — (A, B) Recordings of the ventricular pressure in the masu salmon at 34 mpf. Ventricular and atrial pressures during three heart cycles (A). Ventricular and atrial pressure in the ventricular diastole (B); magnification of the 1 and 2 s range of panel (A). The solid and dotted lines indicate the ventricular pressure and atrial pressure, respectively. VD, ventricular diastole; IR, isovolumic relaxation; AS, atrial systole. (TIF) [file pone.0267264.s004.tif]

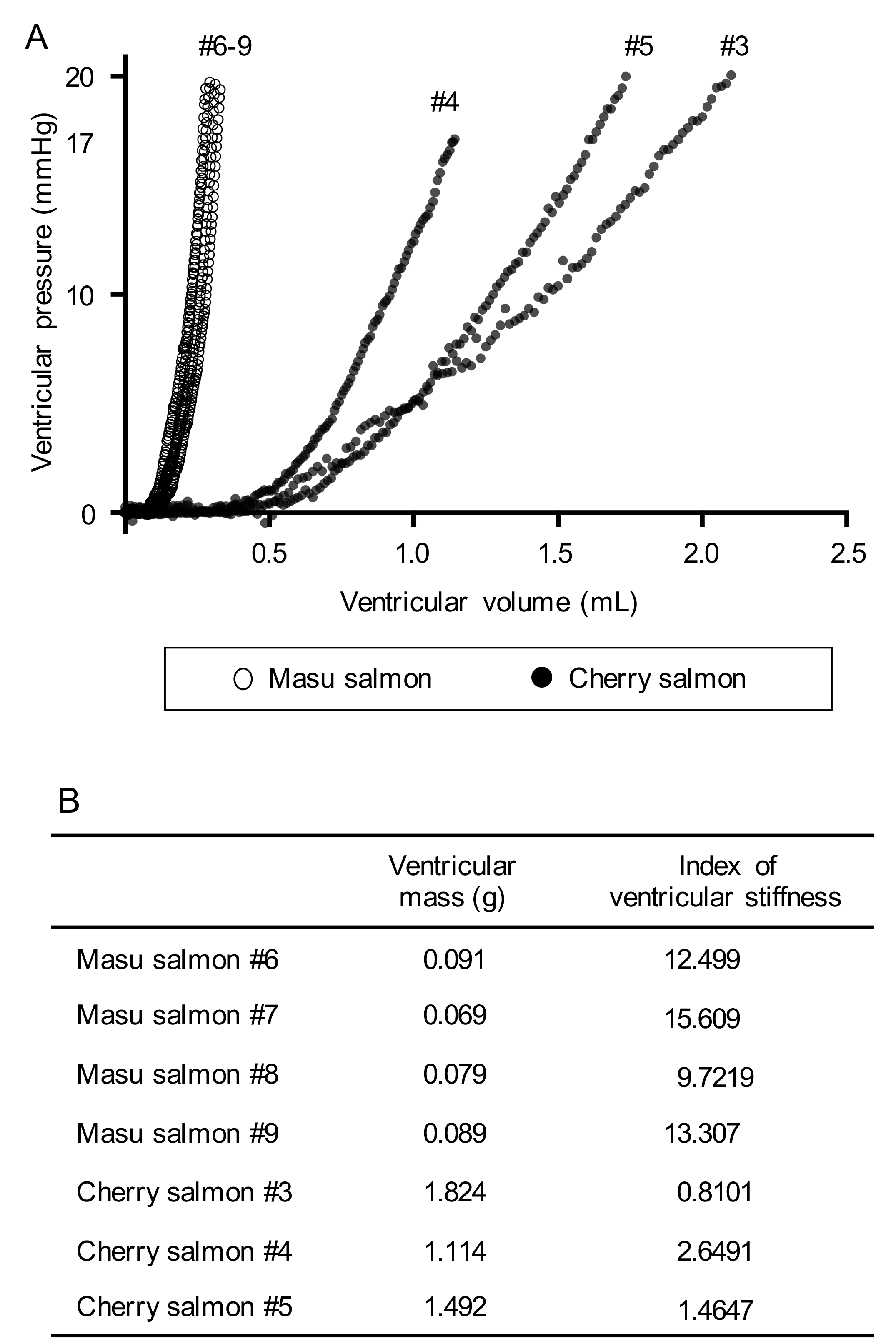

Supplement: S5 Fig — (A) The horizontal axis shows the ventricular chamber volume normalized by the mass of the masu salmon ventricles (N = 4) and the cherry salmon ventricles (N = 3). (B) Ventricular mass and index of ventricular stiffness in the masu and cherry salmon. The results of the EDPVR (A) were applied to Eq (1) to obtain the exponential C, ○: masu salmon, ●: cherry salmon. Actual measurements are shown in the S2 File. (TIF) [file pone.0267264.s005.tif]

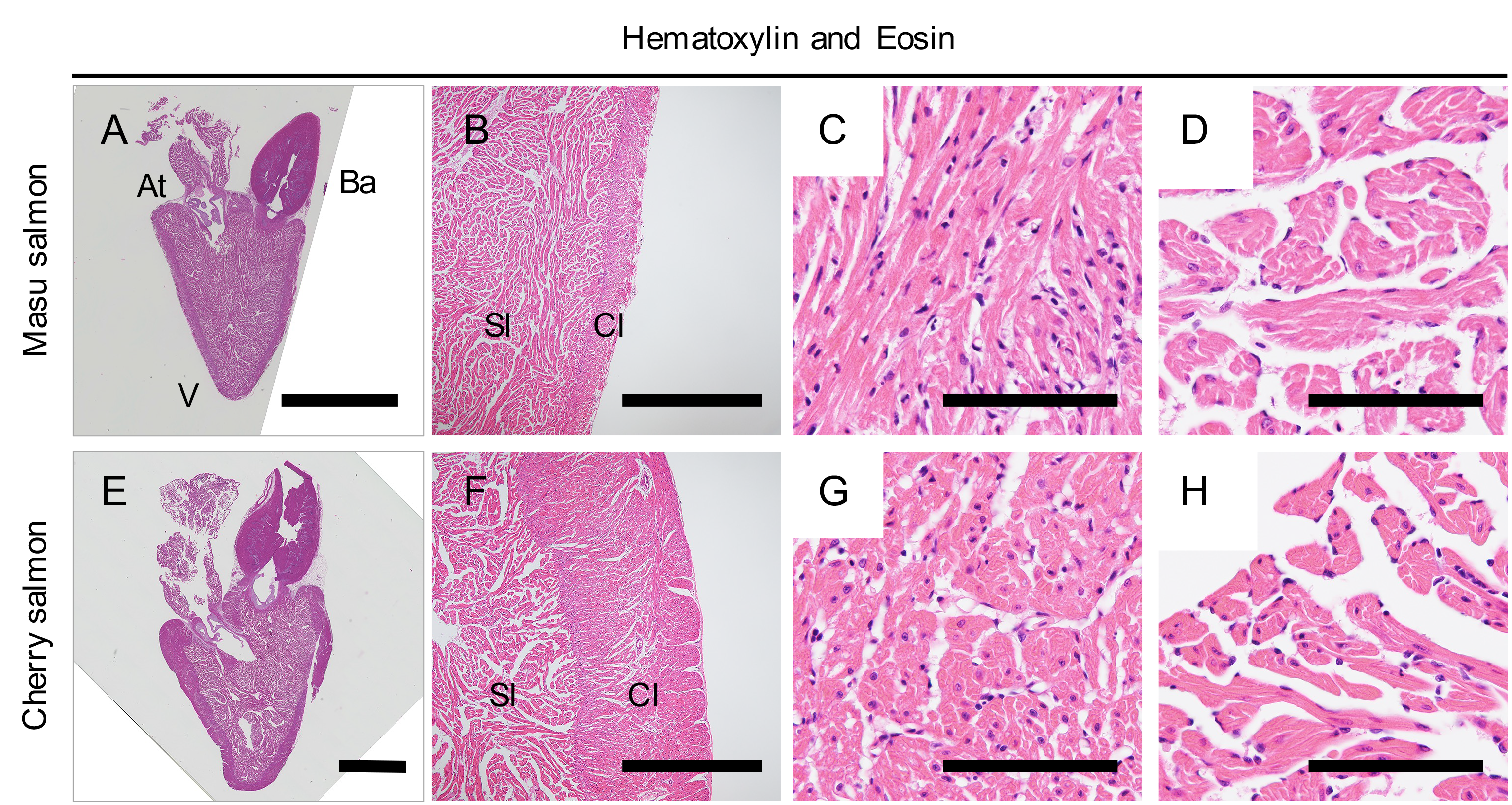

Supplement: S6 Fig — (A–H) Hematoxylin and eosin staining images of the sagittal sections of the hearts at 29 months post fertilization: masu salmon (A–D) and cherry salmon (E–H). (A, E) Images of the whole heart. Scale bars = 5 mm. (B, F) Magnified images of the ventricles. Scale bars = 1 mm. (C, G) High-magnification images of the compact layers. Scale bars = 100 μm. (D, H) High-magnification images of the spongy layers. Scale bars = 100 μm. The cytoplasm is shown in magenta, and the nuclei are shown in blue–purple. At, atrium; V, ventricle; Ba, bulbus arteriosus; Cl, compact layer; Sl, spongy layer. (TIF) [file pone.0267264.s006.tif]

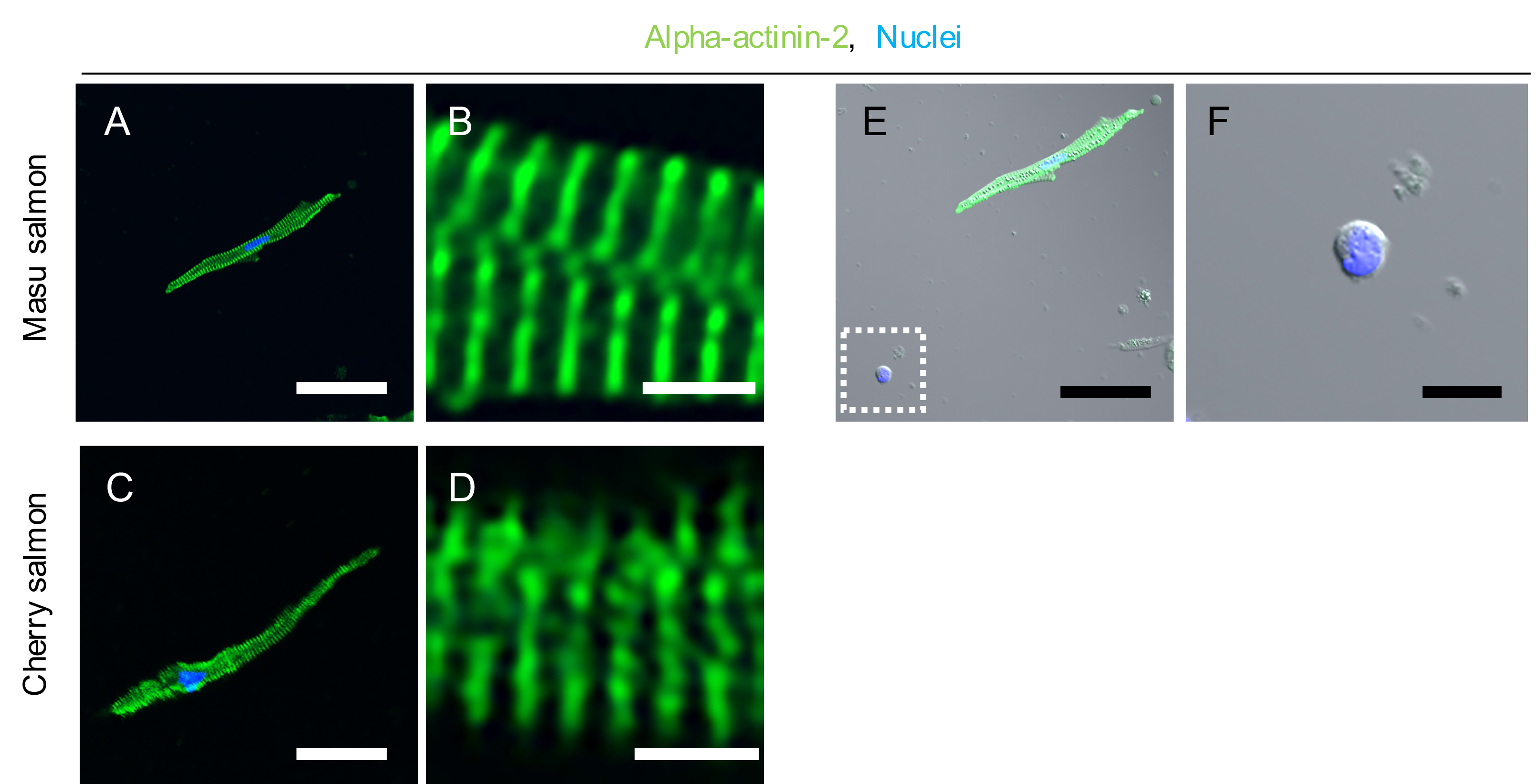

Supplement: S7 Fig — (A–F) Immunofluorescence images show attached cardiomyocytes from the masu and cherry salmon at 29 months post fertilization. Cardiomyocytes were stained with antibodies recognizing Alpha-actinin-2 at z-lines (green) and Hoechst 33342 to detect nuclei (blue). (B, D) Magnifications of the images in (A) and (C). (E, F) Merged bright-field and immunostaining images. (F) Non- cardiomyocyte; magnification view of the white dotted line enclosure in (E). Scale bars = 20 μm in (A), (C) and (E), and 5 μm in (B), (D) and (F). (TIF) [file pone.0267264.s007.tif]

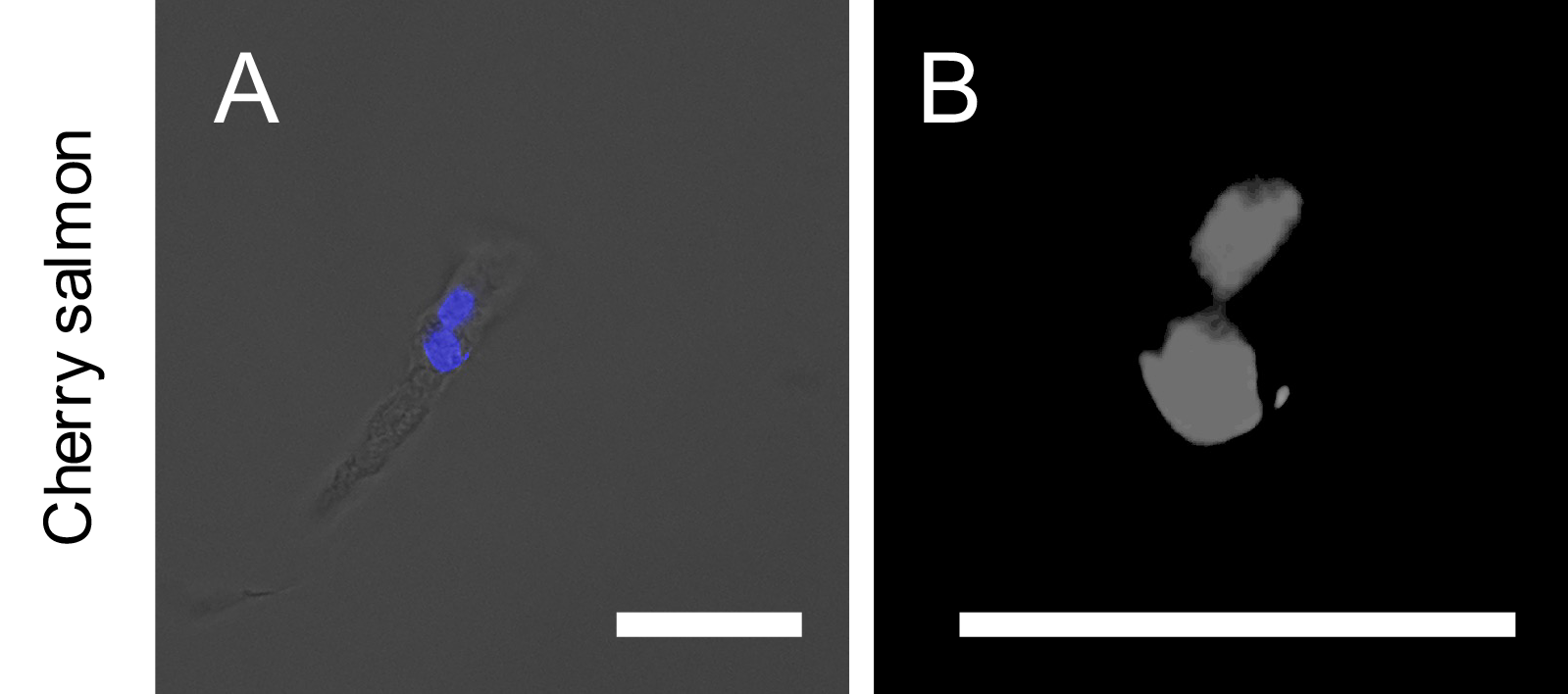

Supplement: S8 Fig — (A) A Hoechst 33342-stained binucleated cardiomyocyte (blue) in a cherry salmon. (B) Magnified image shows Hoechst 33342 signals in the grayscale mode. Scale bars = 20 μm. (TIF) [file pone.0267264.s008.tif]

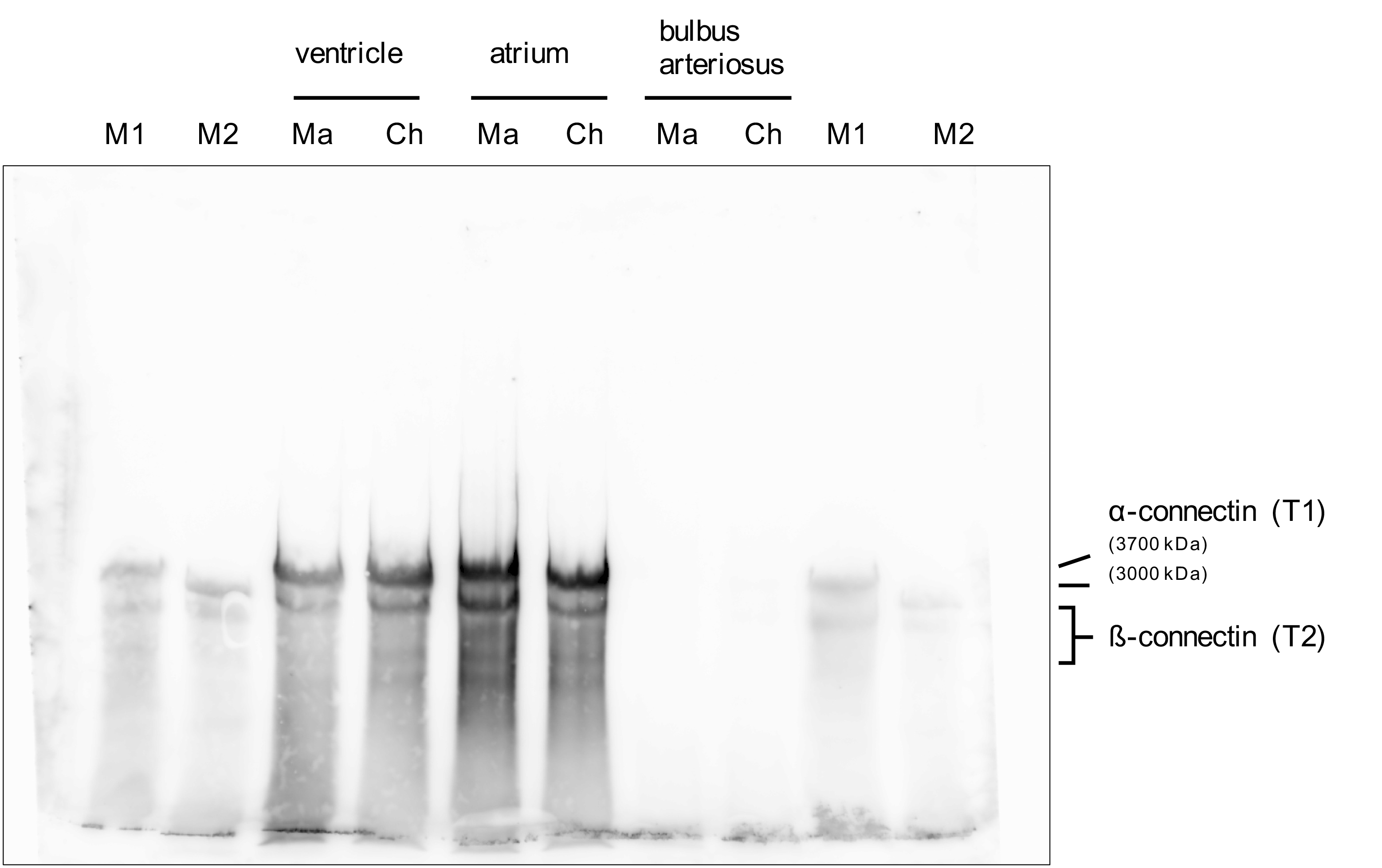

Supplement: S9 Fig — Representative and original immunoblot image showing the detection of Connectin expression in masu (Ma) and cherry (Ch) salmon hearts. Connectin N2A isoform in the mouse skeletal muscle in lanes 1 and 9 (M1, 3,700 kDa) and the N2B isoform in the left ventricle of mice in lanes 2 and 10 (M2, 3,000 kDa) were used as a positive control for the experiment and as a guide for molecular weight. α-connectin (T1) and ß-connectin (T2) indicate intact Connectins and their degraded products, respectively. A raw data image of western blot is found in S1 Raw images. (TIF) [file pone.0267264.s009.tif]

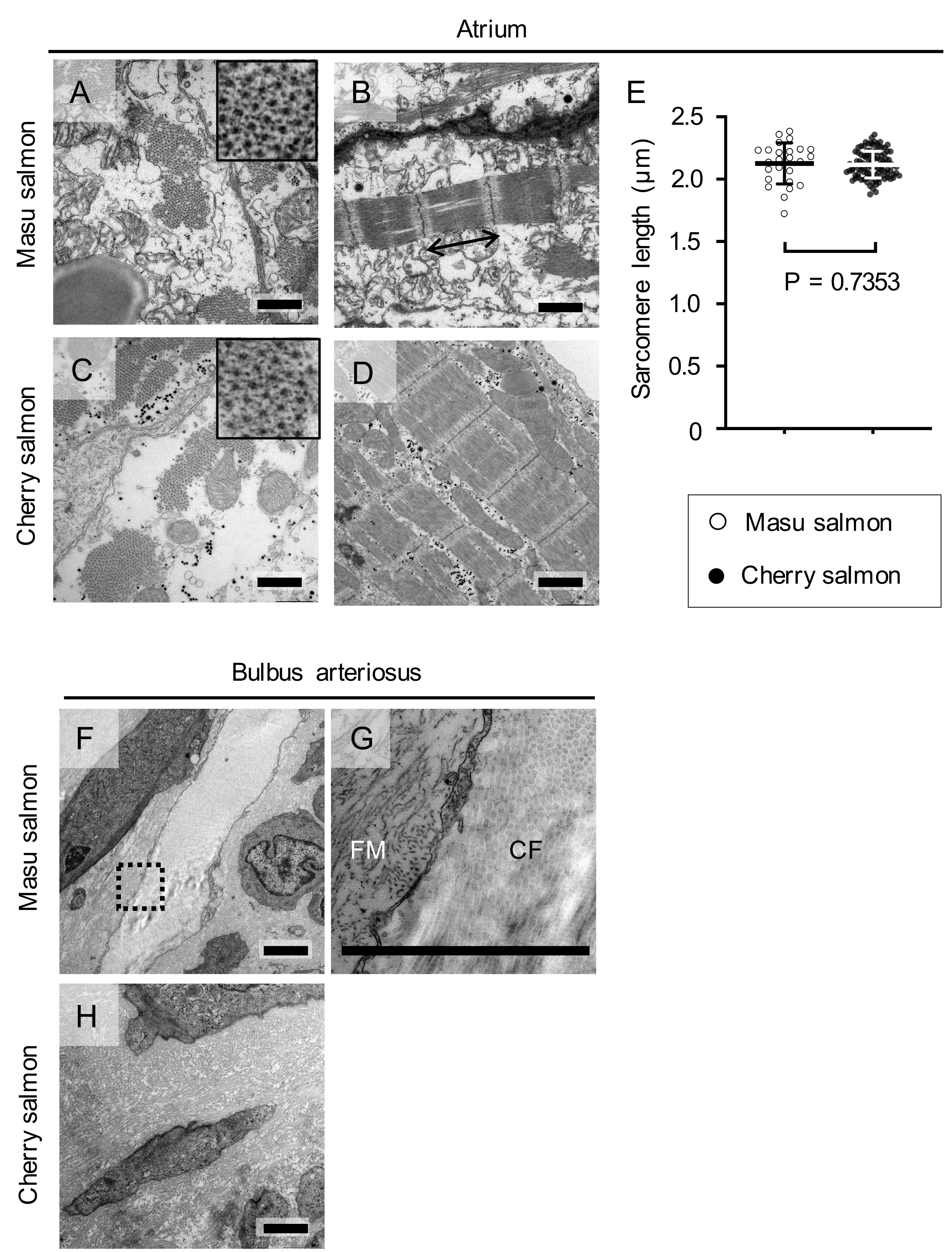

Supplement: S10 Fig — (A–D) Representative transmission electron microscopy images of the cross-sections and longitudinal sections of the atrial sarcomeres of masu and cherry salmon at 29 months post fertilization (mpf). Insets of (A, C) showed higher magnifications of cross-sections of the myofibril bundles. (E) The graph showed atrium sarcomere lengths of the z-line to z-line in masu salmon (2.13 ± 0.16 μm, N = 25) and cherry salmon (2.12 ± 0.11 μm, N = 103). One or two ultrathin slices were observed in each tissue sample. Lines and error bars indicate means ± standard deviations. ○: masu salmon, ●: cherry salmon. (F–H) Representative transmission electron microscopy images of the bulbus arteriosus of masu and cherry salmon at 29 mpf. (G) Higher magnification image in the black dashed line in (F). FM: microfilament; CF: collagen fiber Scale bars = 500 nm in (A, C), 1 μm in (B, D), and 2 μm in (F–H). Minimal data sets for (E) are found in S5 File. (TIF) [file pone.0267264.s010.tif]
