# Supplementary material for: Cardiac hemodynamics and ventricular stiffness of sea-run cherry salmon (Oncorhynchus masou masou) differ critically from those of landlocked masu salmon
Source: PLoS One. 2022 Nov 4;17(11):e0267264. doi: 10.1371/journal.pone.0267264 (PMC9635730; doi:10.1371/journal.pone.0267264)
Supplement: S1 Table — (DOCX) [file pone.0267264.s011.docx]

**S1 Table. Heart rates of the masu and cherry salmon**

| Name | Beat/minute |
| --- | --- |
| Masu salmon #1 | 108 |
| Masu salmon #2 | 126 |
| Masu salmon #3 | 117 |
| Masu salmon #4 | 104 |
| Masu salmon #5 | 91 |
| Cherry Salmon #1 | 77 |
| Cherry Salmon #2 | 67 |

The means ± SDs indicate 109 ± 12.0 bpm in masu salmon and 72 ± 5.3 bpm in cherry salmon. *p* = 0.0164.
