# Supplementary material for: Cardiac hemodynamics and ventricular stiffness of sea-run cherry salmon (Oncorhynchus masou masou) differ critically from those of landlocked masu salmon
Source: PLoS One. 2022 Nov 4;17(11):e0267264. doi: 10.1371/journal.pone.0267264 (PMC9635730; doi:10.1371/journal.pone.0267264)
Supplement: S1 Raw images — (PDF) [file pone.0267264.s019.pdf]

Raw images for Figure 5A

Order of loading:
1. Skeletal muscles of mice
2. Atrium of masu salmon
3. Atrium of cherry salmon
4. Ventricle of masu salmon
5. Ventricle of cherry salmon
6. Bulbus arteriosus of masu salmon
7. Bulbus arteriosus of cherry salmon
8. Zebrafish heart
9. Left ventricle of mice
10. Skeletal muscles of mice

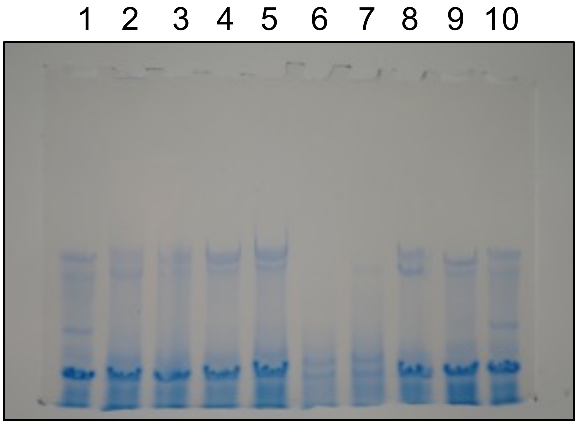

The image was captured using Tough TG-5 4K(OLYMPUS).

Raw images for S9 Fig

Order of loading:
1. Skeletal muscles of mice
2. Left ventricle of mice
3. Atrium of masu salmon
4. Atrium of cherry salmon
5. Ventricle of masu salmon
6. Ventricle of cherry salmon
7. Bulbus arteriosus of masu salmon
8. Bulbus arteriosus of cherry salmon
9. Skeletal muscles of mice
10. Left ventricle of mice

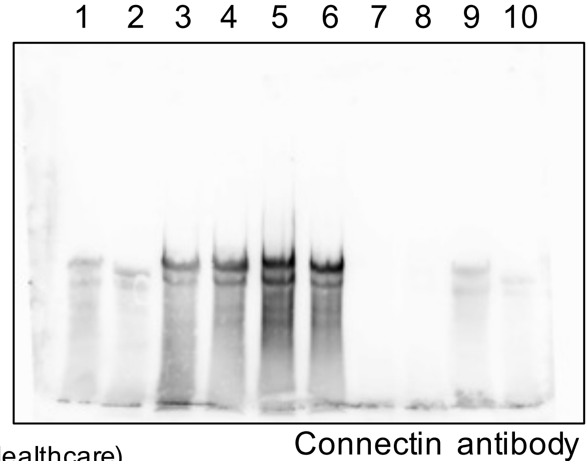

The image was captured using ImageQuant LAS 4000 (GE Healthcare).
